# Novel *Pseudomonas* sp. SCA7 Promotes Plant Growth in Two Plant Families and Induces Systemic Resistance in *Arabidopsis thaliana*

**DOI:** 10.3389/fmicb.2022.923515

**Published:** 2022-06-27

**Authors:** Theresa Kuhl-Nagel, Patricia Antonia Rodriguez, Isabella Gantner, Soumitra Paul Chowdhury, Patrick Schwehn, Maaria Rosenkranz, Baris Weber, Jörg-Peter Schnitzler, Susanne Kublik, Michael Schloter, Michael Rothballer, Pascal Falter-Braun

**Affiliations:** ^1^Institute for Network Biology, Helmholtz Center Munich, German Research Center for Environmental Health (GmbH), Neuherberg, Germany; ^2^Microbe-Host Interactions, Faculty of Biology, Ludwig-Maximilians-University of Munich, Munich, Germany; ^3^Institute of Biochemical Plant Pathology, Research Unit Environmental Simulation, Helmholtz Center Munich, German Research Center for Environmental Health (GmbH), Neuherberg, Germany; ^4^Research Unit for Comparative Microbiome Analysis (COMI), Helmholtz Center Munich, German Research Center for Environmental Health (GmbH), Neuherberg, Germany

**Keywords:** plant-microbe interactions, PGPB, *Arabidopsis thaliana*, *Triticum aestivum* L., *Pseudomonas*, biocontrol, ISR, mVOCs

## Abstract

*Pseudomonas* sp. SCA7, characterized in this study, was isolated from roots of the bread wheat *Triticum aestivum*. Sequencing and annotation of the complete SCA7 genome revealed that it represents a potential new *Pseudomonas* sp. with a remarkable repertoire of plant beneficial functions. *In vitro* and *in planta* experiments with the reference dicot plant *A. thaliana* and the original monocot host *T. aestivum* were conducted to identify the functional properties of SCA7. The isolate was able to colonize roots, modify root architecture, and promote growth in *A. thaliana*. Moreover, the isolate increased plant fresh weight in *T. aestivum* under unchallenged conditions. Gene expression analysis of SCA7-inoculated *A. thaliana* indicated a role of SCA7 in nutrient uptake and priming of plants. Moreover, confrontational assays of SCA7 with fungal and bacterial plant pathogens revealed growth restriction of the pathogens by SCA7 in direct as well as indirect contact. The latter indicated involvement of microbial volatile organic compounds (mVOCs) in this interaction. Gas chromatography-mass spectrometry (GC-MS) analyses revealed 1-undecene as the major mVOC, and octanal and 1,4-undecadiene as minor abundant compounds in the emission pattern of SCA7. Additionally, SCA7 enhanced resistance of *A. thaliana* against infection with the plant pathogen *Pseudomonas syringae pv. tomato* DC3000. In line with these results, SA- and JA/ET-related gene expression in *A. thaliana* during infection with *Pst* DC3000 was upregulated upon treatment with SCA7, indicating the ability of SCA7 to induce systemic resistance. The thorough characterization of the novel *Pseudomonas* sp. SCA7 showed a remarkable genomic and functional potential of plant beneficial traits, rendering it a promising candidate for application as a biocontrol or a biostimulation agent.

## Introduction

Agroecosystems are under increasing pressure by soil exploitation, intensive use of mineral fertilizers and pesticides, as well as climate change (Foley et al., 2005; Tsiafouli et al., 2015; Smith et al., 2016). Plants exposed to abiotic stresses are more susceptible to pests and pathogens. At the same time, increasing temperatures and elevated global traffic enable pathogens to expand beyond their original habitat and infect new host plants (Bebber et al., 2014). Up to one third of the global harvest is lost due to plant diseases (Savary et al., 2019). Moreover, pathogens evolve quickly and can overcome plant defense systems and known pesticides, which might reduce the effectiveness of plant pathogen control measures (McDonald and Stukenbrock, 2016). However, excessive use of chemical pesticides is harmful to insects and also to humans (Uhl and Brühl, 2019; Hirt, 2020). Therefore, the situation warrants a constant need for discovering and developing new sustainable plant growth-promoting and biocontrol solutions, which protect plants and support environmentally friendly agriculture.

Promising approaches involve exploiting the plant root microbiome and, here, especially the plant-growth promoting (rhizo-) bacteria (PGPR or PGPB), originally defined as “free-living plant beneficial bacteria, which promote plant health” (Kloepper and Schroth, 1978). Underlying plant beneficial mechanisms of PGPB associated with plant roots include, e.g., the stimulation of plant growth *via* production of plant hormones, such as auxin (indoleacetic acid, IAA) or nutrient supply *via* siderophore production of iron, solubilization of inaccessible mineral phosphate or biological nitrogen fixation (Glick, 2012). The beneficial associations are of special importance when plants experience abiotic or biotic stresses (Fahad et al., 2015). For example, under pathogen attack, PGPB can support the plant *via* the production of active biomolecules, such as antibiotics, lipopeptides, and polyketides (Chowdhury et al., 2015) or volatile compounds (Netzker et al., 2020) against pathogenic bacteria and fungi. PGPB can also act indirectly by competition with other microbes for iron *via* production of siderophores (Gu et al., 2020) or by inducing host systemic resistance (ISR) (Kloepper and Beauchamp, 1992; Pieterse et al., 2014). Additionally, bacteria can help to balance the plant microbiome or decrease detrimental effects of pathogenic fungi (Duran et al., 2018). Plants and their associated microbes form a functional entity (Vandenkoornhuyse et al., 2015), although each member of the microbiota is an individual organism with its own functions (Marín et al., 2021). Important functional insights into plant beneficial bacteria are often derived from experiments with a reductionist approach using culturable, well-characterized single strains (Schlaeppi and Bulgarelli, 2015), such as *Pseudomonas simiae* WCS417 (Pieterse et al., 2020). Although several studies have elucidated the underlying molecular mechanisms involved in various plant-microbe interactions, much remains to be understood to exploit the full potential of plant beneficial traits for sustainable agriculture (Berg et al., 2017; Rodriguez et al., 2019; Babin et al., 2021; Windisch et al., 2021).

Representatives of the genus *Pseudomonas* are important members of the plant holobiont at the plant soil interface (Bakker et al., 2013). The genus comprises rod-shaped, Gram-negative bacteria with a polar flagellum and includes some of the best known PGPB (Pieterse et al., 2014). One example is the intensively studied *Pseudomonas simiae* WCS417, which enhances plant growth and increases lateral root formation in the model plant *Arabidopsis thaliana* (Pieterse et al., 2020). Furthermore, *Pseudomonas fluorescens* SS101 can increase *Nicotiana tabacum* biomass *via* production of mVOCs (Park et al., 2015). Other plant-associated *Pseudomonas* species have been shown to be active against various plant pathogens. For example, *Pseudomonas chlororaphis* MA 432 (Johnsson et al., 1998) protects the cereal plants barley, oats, wheat, and rye against several plant-pathogenic fungi and is part of a commercial seed coverage biocontrol product [EFSA (European Food Safety Authority), 2017]. Moreover, *Pseudomonas fluorescens* WR-1 restricts fungal growth of *Ralstonia solanacearum* on tomato *via* mVOC emissions (Raza et al., 2016). Bioactive compounds, such as 2,4-diacetylphloroglucinol and phenazine, a common antifungal secondary metabolite produced by a number of strains of the genus *Pseudomonas* (Mavrodi et al., 2006; Yu et al., 2018; Tagele et al., 2019), as well as mVOCs (Kai et al., 2009; Raza et al., 2016; Netzker et al., 2020), have been shown to be responsible for the antimicrobial activities of various *Pseudomonas* strains. On the other hand, some plant-associated *Pseudomonas* species are able to cause plant diseases, such as *Pseudomonas syringae* (Xin and He, 2013), which has a broad host range and mainly infects aerial parts of the host. The variant *Pseudomonas syringae* pv. *tomato* strain DC3000 (*Pst* DC3000) colonizes successfully *A. thaliana* plants, and, together, they form a model interaction system for studying plant disease resistance (Xin and He, 2013). *Pst* DC3000 invades host cells by suppressing basal and effector-triggered immunity (ETI) responses through translocation of type III secretion system (T3SS)- effector proteins, such as AvrPto, AvrRpt2, and AvrRpm1, (Xin and He, 2013). Additionally, *Pst* DC3000 produces coronatine that suppresses salicylic acid (SA) signaling pathways during immunity, facilitating the colonization process (Xin and He, 2013). Interestingly, other *P. syringae* strains have been reported as plant beneficial, such as *P. syringae pv. syringae* 260-02 (Passera et al., 2019). Thus, members of the genus *Pseudomonas* have a diverse range of functions, and even members of the same species can act as plant growth promoters, as antagonists toward plant pathogens, but also as plant pathogens themselves. A close phylogenetic relationship to a pathogen could hamper the use of a new potentially beneficial isolate in both experimental and commercial applications. On the one hand, high genetic similarity of a beneficial and a pathogenic strain bears the risk of also sharing similar virulence genes, and, on the other hand, the acceptance of a beneficial strain with the same species name as a known pathogen might hamper the acceptance of this new beneficial by the customers. Therefore, the phylogenetic separation from pathogens is an important first step in the characterization of a new potentially beneficial isolate. A common approach to quickly identify bacteria is based on the analysis of 16S rRNA gene sequences (Janda and Abbott, 2007). However, due to the limited phylogenetic resolution provided by the 16S rRNA gene, an unambiguous classification of new isolates solely based on the small ribosomal subunit might not be sufficient (Hartmann et al., 2019). Thus, often, complete genome sequencing is used to discriminate between beneficial and pathogenic bacterial strains, allowing mostly reliable identification and assignment of new isolates. For example, whole genome sequencing has been successfully used to allow the phylogenetic separation of the opportunistic human pathogen *Roseomonas fauriae* from the plant beneficial *Azospirillum brasilense*, which were previously allocated to the same species *A. brasilense* (Levy et al., 2018; Hartmann et al., 2019). Nevertheless, even the genome analysis cannot replace the experimental characterization of actual functional traits.

In this work, we aimed to characterize the PGP and biocontrol abilities of *Pseudomonas* sp. SCA7, a novel isolate from a bacterial rhizosphere culture collection of wheat (*Triticum aestivum* L.) cultivated in an agricultural field. By using whole genome sequencing, we could assign a taxonomic position to SCA7 in the complex *Pseudomonas* phylogeny, discriminate it from pathogens, and elucidate its plant beneficial capabilities. The goal was to identify and validate PGP and antagonistic functional traits of SCA7 by *in vitro* and *in planta* approaches using the model organism *A. thaliana* and the original host *T. aestivum* plants. A special focus was on the identification of mVOCs produced by the isolate, which could be involved in the observed effects.

## Materials and Methods

### Isolation of *Pseudomonas* sp. SCA7

*Pseudomonas* sp. SCA7 (= starch casein agar, colony 7), named hereafter SCA7, was isolated from roots and rhizosphere of bread wheat plants (*Triticum aestivum*, cultivar “Sonett”) short before harvest grown on an agricultural field of a long-term field trial (since 1990), with flat tillage from the research farm of Helmholtz Munich in Scheyern, Bavaria, Germany (48.50029, 11.44501) in July 2018. The soil has been classified as luvisol, with a sandy and loamy texture (43% sand, 33% silt, and 24% clay) (Yang et al., 2020). Roots were shaken to remove the loosely adhering soils and were grounded with a 1 x phosphate saline buffer (PBS). The rhizosphere soil extract was diluted three times, plated on starch casein agar [SCA: 10 g/L starch, 0.3 g/L casein, 2 g/L potassium nitrate (KNO_3_), 2 g/L sodium chloride (NaCl), 2 g/L K_2_HPO_4_, 0.05 g/L ${\text{MgSO}}_{4}^{*}$7H2O, 0.02 g/L calcium carbonate (CaCO_3_), 0.01 g iron sulfate (${\text{FeSO}}_{4}^{*}$7H2O)] and allowed to grow at 22°C for 3 days. Emerging colonies appeared in beige color with a smooth and shiny surface. Colonies were picked and allowed to grow on nutrient broth (NB) agar at 28°C. For SCA7, an optical density (OD_600_) at 600 nm of 0.1 corresponds to approximately 2 × 10^7^ CFU/ml. The strain was selected for further characterization based on a pre-screening of isolates for plant growth-promoting properties *in vitro*, where it showed siderophore and IAA production as described below. Additionally, the isolate SCA7 belongs to the genus *Pseudomonas*, which harbors many strains with biocontrol potential and inhibited growth of 11 other bacterial isolates from wheat rhizosphere, belonging to the genera *Bacillus, Dyadobacter, Flavobacterium, Luteibacter, Rhizobium, Pseudomonas, Sphingomonas*, and *Variovorax* in a pairwise interaction assay, which raised interest in characterizing the potential antagonistic activities of SCA7.

### Whole Genome-Based Analysis

#### Genome Sequencing

DNA of SCA7 was isolated with DNeasy^®^UltraClean^®^Microbial Kit (Qiagen, Hagen, Germany), following the manufacturer's protocol. Microbial Library was prepared for PacBio Sequel system using SMRTbell^®^ Express Template Prep Kit 2.0 and Barcoded Overhang Adapter Kit 8A, following the PacBio instruction “Procedure & Checklist- Preparing Multiplexed Microbial Libraries Using SMRTbell^®^ Express Template Prep Kit 2.0.” PacBio sequencing was performed with the Sequel sequencing kit 3.0 (4 reactions) and a single-molecule real-time (SMRT) cell 1 M v3 tray. Sequencing was performed on the PacBio Sequel system, with 120-min immobilization and 120-min pre-extension time, followed by 600-min movie time. The SCA7 genome was assembled using the Microbial assembly pipeline of the single molecule real-time (SMRT) portal interface (v9.0 PacBio SMRTLink^®^, Pacific Biosciences), with default parameters and internal quality check.

#### Phylogenetic Analysis

Closely related species to SCA7 based on 16S rRNA gene were identified using the Software ARB (5.3). In the Type Strain Genome Server (TYGS) (Meier-Kolthoff and Göker, 2019), closely related type strains based on digital DNA-DNA hybridization (dDDH) of the full genome sequence of SCA7 were identified. The full-genome maximum-likelihood phylogenetic tree build in EDGAR 3.0 (Blom et al., 2016) was based on 307 genomes of the genus *Pseudomonas* and two genomes of *Herbaspirillum frisingense* IAC152, as well as *Streptomyces albus* NBRC 13014 (type strain) as outgroups using FastTree Software with the Shimodaira-Hasegawa test for bootstrap values. A subtree, including 11 closely related type strains and *H. frisingense* IAC152 as an outgroup, was calculated with EDGAR 3.0 (Blom et al., 2016). Two average nucleotide identity (ANI) matrices were calculated in EDGAR 3.0 (Blom et al., 2016) as described in Goris et al. (2007) and Konstantinidis and Tiedje (2005). The first was based on genomes of 11 *Pseudomonas* strains closely related to SCA7; the second was based on genomes of type strains of 11 *Pseudomonas* species closely related to SCA7.

#### Functional Annotation of SCA7 Genome

The SCA7 genome was annotated using the NCBI prokaryotic Genome Annotation Pipeline (PGAP) and the Rapid Annotation using Subsystem Technology 2.0 (RAST) and browsed afterward in the SEED environment (Aziz et al., 2008; Overbeek et al., 2014). Although based on different algorithms, both annotation programs correctly identify more than 95% of the genes present in a bacterial genome (Berrios and Ely, 2018). Further functional annotation was performed by identifying gene clusters for biosynthesis of secondary metabolites with the antibiotics and the secondary metabolite analysis shell—antiSMASH 6.0 (Blin et al., 2017) using default parameters, using CARD – The Comprehensive Antibiotic Resistance Database (Version 2020; Alcock et al., 2020) for identification of antibiotic resistance genes and VFDB—The virulence factor database (Version 2019; Liu et al., 2019).

### *In vitro* and *in planta* Analyses of Bacterial Traits

#### Cultivation and Growth Conditions of Microorganisms

In the experiments conducted in the study, the following bacterial strains were used as control or as plant pathogenic strains: the strain *Bacillus velezensis* FZB42 DSM 23117, producing antifungal compounds like surfactin, fengycin, and iturin (Chowdhury et al., 2015), the IAA-producing *Herbaspirillum frisingense* GSF30 DSM 1328 (Kirchhof et al., 2001), the siderophore-producing *Rhodococcus qingshengii* RL1 (Kuhl et al., 2021), the AHL biosensor strain *Agrobacterium tumefaciens* A136 ATCC 51350 (Han et al., 2016), the AHL-producer strain *Acidovorax radicis* N35 DSM 23535 (Li D. et al., 2011), the biofilm-producing *Pseudomonas simiae* WCS417r (Pieterse et al., 2020), and the non-biofilm-producing *Escherichia coli* DH5α (Anton and Raleigh, 2016). Plant-pathogenic strains were *Xanthomonas translucens* (Sapkota et al., 2020) and *Pseudomonas syringae* DC3000 (*Pst* DC3000) (Xin and He, 2013). Bacterial strains were cultivated in liquid nutrient broth (NB, Roth) (beef extract, 3 g/L; gelatin peptone, 5 g/L) or solid nutrient agar (with 1.7% agar), with pH 6.8, unless further specified, at 30°C at 130 rpm. *Pst* DC3000 was cultivated in nutrient yeast glycerol [NYG: 5 g/L bacto-peptone (BD Difco^TM^)], 3 g/L yeast extract (Roth), a 20 ml/L glycerol (Roth, pH 7) medium, supplemented with rifampicin (50 μg/L), and kanamycin (30 μg/L). The plant-pathogenic fungi *Rhizoctonia solani*, causing potato stem cancer and black scurf (Yang and Li, 2012); wheat pathogenic fungus *Fusarium culmorum* G2191, causing seedling blight, foot rot, and head blight; (Wagacha and Muthomi, 2007) and the wilt-causing *Fusarium oxysporum* DSM62297 (Gerlach et al., 1958) were used in confrontation experiments with SCA7. Fungi were cultivated on potato dextrose agar (PDA, Sigma) (potato extract, 4. g/L; glucose, 20. g/L) at room temperature in the dark and stored at 4°C until further use.

#### Swarming Motility

Swarming motility was analyzed according to Caiazza et al. (2005) with modifications. Overnight grown cultures were washed two times in 1 x PBS and diluted to 2 × 10^7^ CFU/ml using 1 x PBS. One microliter of the washed bacterial culture was pipetted on plates with an M9 minimal medium (33.1 mM Na_2_HPO_4_, 22 mM KH_2_PO_4_, 8.55 mM NaCl, 9.35 mM NH_4_Cl, 0.4% glucose (w/v), 1 mM MgSO_4_, 0.3 mM CaCl_2_), supplemented with 0.5% casamino acids (w/v) and 0.5% (w/v) agar. Swarming motility was evaluated after 18 h of incubation at 30°C. *E. coli* DH5α served as negative control. The assay was performed in duplicate and repeated three times.

#### Biofilm Formation

Biofilm formation was analyzed following the protocol of O'Toole (2011). Briefly, an overnight grown culture was washed two times with 1 x PBS and centrifuged. The pellet was resuspended with the M9 minimal medium, supplemented with 0.5% casamino acids and adjusted to 2 × 10^7^ CFU/ml. A total of 100 μL of resuspended bacterial culture was transferred to a flat-bottom 96 well microtiter plate and incubated for 24 h at 30°C. Growth was determined by optical density (SpectraMax iD3, Molecular Devices) at 600 nm. Unattached cells were removed from the plate by gently washing the wells two times with distilled water. Subsequently, 125 μl of 0.1% crystal violet solution was added to each well and incubated for 10 min at room temperature. After washing three times with water, the plate was dried for 2 h. Biofilm formation was assessed by formation of a ring-shaped violet stained biofilm matrix, and was documented by photographs. The stained biofilm was solubilized with 125 μl of 30% acetic acid and incubated for 10 min at room temperature. The solution was transferred to a new microtiter plate, and matrix intensity was quantified by optical density (SpectraMax iD3, Molecular Devices), with absorbance at 550 nm and acetic acid served as a blank. The experiments were performed with twelve replicates per strain. Quantification was based on the biofilm intensity normalized with OD_600_ = 1. *P. simiae* WCS417r served as positive control, and *E. coli* DH5α, as well as a medium without cells, served as negative control.

#### N-Acyl Homoserine Lactone (AHL) Production

N-acyl homoserine lactone (AHL) production was analyzed using the biosensor assay as described in Han et al. (2016). Briefly, a single colony of the biosensor strain *A. tumefaciens* A136 was picked and streaked in a vertical line on a fresh NB plate, containing 40 μg/ml X-Gal. Single colonies of SCA7 and the positive control *A. radicis* N35 were streaked horizontally and close to the biosensor strain. After incubation for 24 h at 28°C, AHL production was detected by blue-color formation at the contact zone of test and biosensor strain caused by the activation of the reporter fusion *traI-lacZ* and X-Gal metabolization.

#### Indole-Acetic Acid (IAA) Production

Determination of IAA production was performed according to the method of Gordon and Weber (1951), as described in Kuhl et al. (2021), with modifications. A pre-grown liquid culture of SCA7 was transferred to a fresh NB medium supplemented with 5 mM tryptophan (1 mg/ml, Sigma) and grown for 24 h. Afterward, liquid cultures were centrifuged, and the collected supernatant was mixed in equal volumes (100 μL) with Salkowski reagent [0.01 M FeCl3 anhydrous (Fluka Biochemika) in perchloric acid (HClO4) 35% (Merck)] (Loper and Schroth, 1986) and 1 μL of orthophosphoric acid (Sigma). After 30 min incubation in the dark, the amount of IAA in the supernatant was analyzed in a plate reader (Spectra Max iD3, Molecular Devices) at 530 nm. A standard curve, with concentrations ranging from 0 to 100 μg/ml, was prepared from commercial indole-3-acetic acid (Fluka Biochemika, Buchs, Switzerland) in NB. Measurements were performed in triplicate, and *H. frisingense* GSF30 served as positive control. IAA production was quantified based on the amount of produced IAA normalized to OD_600_ = 1.

#### Siderophore Production

Analysis of siderophore production was performed following the protocols of Pérez-Miranda et al. (2007) and Lynne et al. (2011), with modifications as described in Kuhl et al. (2021). Briefly, 25 μL of an SCA7 overnight-grown culture was pipetted on NB agar plates and incubated for 48 h. Dye solutions [chrome azurol blue S (Sigma), FeCl3 (Fluka Biochemika) and HDTMA (Hexadecyltrimethylammonium bromide, Sigma)] were prepared and mixed according to Lynne et al. (2011). Piperazin-N,N'-bis-(2-ethanesulfonic acid) (Pipes, Roth) solution was prepared with 0.9% agar and pH 6.8. The dye solutions were autoclaved separately and then slowly mixed with the Pipes-Agar mix. Cooled but still liquid overlay agar (10 ml) was poured on plates with bacteria. Color change from blue to orange after 2 h incubation indicated siderophore production. Siderophore-producing *R. qingshengii* RL1 was used as positive control. The experiment was performed three times.

#### Confrontation Assays Against Plant-Pathogenic Microorganisms

SCA7 was tested for its antagonistic activity *in vitro* against the pathogenic bacteria *X. translucens* and *Pst* DC3000 and the pathogenic fungi *Rhizoctonia solani, Fusarium culmorum* G2191, and *Fusarium oxysporum* DSM62297, as well as the plant beneficial strain *B. velezensis* FZB42. Bacterial strains were pre-grown in NB. *Pst* DC3000 was grown in NYG, supplemented with antibiotics. Overnight-grown cultures were washed two times in 1 x PBS and diluted with fresh NB to 2 x 10^7^ CFU/ml. Antagonistic activity against microorganisms was analyzed in two versions using petri dishes with NB agar for direct contact or split plate dishes with half NB and half PD agar for indirect contact. For confrontation assays against bacteria, bacterial strains were plated with v-shape by pipetting eight times 1 μL of washed bacterial culture in a diagonal line with decreasing horizontal distance between the tested strains. For confrontation assays against fungi, approximately 2 mm^3^ of fungal mycelium was transferred aseptically from a freshly grown plate onto testing plates. For direct contact, 10 μL of washed bacterial solution of SCA7 was placed in approximately 3 cm distance to fungal pieces on the agar plate. For indirect contact, eight times 1 μL of bacterial solution was pipetted in a diagonal line on a split plate. Plates were incubated at room temperature for fungi or at 30°C for bacteria and evaluated after 7 days. The experiments were performed in triplicate.

#### Microbial VOC Detection With Gas Chromatography-Mass Spectrometry (GC-MS)

For mVOCs analysis, SCA7 was pre-grown in NB. Overnight-grown cultures were diluted with fresh NB to 2 x 10^7^ CFU/ml, and 16 times 1 μL was pipetted with V-shape onto NB agar (20 ml) glass petri dishes. The experiment was performed with 10 replicate plates per treatment, and a pure NB medium served as negative control. mVOCs were collected by headspace sorptive extraction (HSSE) using the stir bar sorptive extraction (SBSE) method with Gerstel Twisters^®^ (Gerstel GmbH & Co. KG, Mülheim an der Ruhr, Germany) as previously described (Müller et al., 2013; Guo et al., 2019). The twisters with polydimethylsiloxane (PDMS) coating (film thickness: 0.5 mm, length: 10 mm) were attached to the interior of the glass petri dish lid. The plates were sealed with Parafilm and incubated at 30°C. mVOCs from the Petri dishes' headspace were passively adsorbed on the PDMS film for a period of 24 h or 48 h (*n* = 5 for each), respectively. The samples were analyzed with a thermo-desorption unit (Gerstel) coupled to a gas chromatograph-mass spectrometer (GC–MS; a GC model: 7890A; an MS model: 5975C; Agilent Technologies, Santa Clara, CA, USA), following established procedures (Guo et al., 2019, 2020). δ-2-Carene (860 pmol/μL) served as an internal standard. Compounds were separated using a capillary GC column [(14%-Cyanopropyl-phenyl)-methylpolysiloxane, 70 m x 250 μm; film thickness:0.25 μm]. The carrier gas was helium with a constant flow rate of 1.2 ml/min. The GC oven was preheated to 40°C, and samples were treated using the following temperature cycle: First, temperature was increased to 130°C at a rate of 10°C /min and held for 5 min, and then to 175°C at a rate of 80°C/min, and then to 200°C at a rate of 2°C /min; afterward, to 220°C at a rate of 4°C/min and, finally, to 300°C at a rate of 100°C/min where the temperature was held for another 6 min. The chromatograms were analyzed, employing the software MSD ChemStation E 02.01 1177. Compounds with a low frequency (= present less than in three biological replicates) were discarded from the data set, similar to the VOCs that were released by the sampling system (i.e., glass petri dishes, medium, and Twisters). The total ion count (TIC) of each compound was recalculated from the absolute abundance of the first representative m/z to eliminate chromatogram noise. Annotation was performed by comparison of the mass spectra against libraries of reference spectra (NIST 20, Wiley 275) and non-isothermal Kovats retention indices found in the literature. The retention indices were calculated according to Van Den Dool and Kratz (1963) and are presented in Supplementary Table S7.

#### Seed Surface Sterilization

*A. thaliana* seeds were surface sterilized with 75% ethanol for 4 min and centrifuged for 1 min at 11,000 *g*. These steps were followed by 100% ethanol incubation, centrifugation for 5 min at 11,000 x g and afterwards seeds were stratified at 4°C in the dark for 3 days. *T. aestivum* seeds were placed on moistened filter papers and stored overnight at 4°C. After 12 h, *T. aestivum* seeds were surface sterilized by washing in 1% (w/v) Tween-80 for 2 min and incubated in 12% (w/v) NaOCl for 7 min. The seeds were washed 3 x with sterile water and mixed in an antibiotic solution [100 ml distilled water, 1 ml Penicillin-G (60 mg/ml) and 100 μL Streptomycin (250 mg/ml)] for 10 min. After discarding the supernatant, the seeds were transferred to Hoagland's solution agar plates and moistened with a drop of sterile water. For germination, *T. aestivum* seeds were incubated at room temperature in the dark for 3 days.

#### SCA7 Growth-Promoting Activity on *A. thaliana* in Axenic Conditions

Surface-sterilized and stratified seeds of *A. thaliana* were placed on ½ MS agar (2.16 g/L Murashige Skoog (MS), including B5 vitamins (Duchefa Biochemie, Haarlem, The Netherlands), 8 g/L plant agar (Duchefa Biochemie), adjusted to pH 5.7. Plants were placed in a growth chamber (Weiss Technik, Modell SGC120PG2, Reiskirchen, Germany) at 23°C, 55 % humidity, and, approximately, 80 μmol photons m^−2^ s^−1^ of photosynthetic active radiation (PAR) with a 12 h light−12 h dark interval. After 7 days, seedlings with an equal root length of about 1.5 cm were transferred to new MS plates, following the protocol of Asari et al. (2017). Overnight-grown cultures of SCA7 were washed two times in 1 x PBS and adjusted to 2 × 10^7^ CFU/ml according to optical density. To determine this optimal cell concentration for the upcoming experiments, three different cell concentrations (2 x 10^7^ CFU/ml, 2 x 10^5^ CFU/ml, 2 x 10^3^ CFU/ml) were tested in pre-experiments, which all showed a beneficial effect on root biomass, with the strongest effect on the concentration 2 x 10^7^ CFU/ml. Therefore, the highest cell concentration of 2 x 10^7^ CFU/ml was used for the main experiments. Approximately, 10 μL of the bacterial solutions was pipetted at a distance of 1 cm from the root tip of the transferred plants. One x PBS and heat-killed (= autoclaved) bacteria were used as mock and negative controls, respectively. The plates were sealed with micropore tape and placed vertically in a growth chamber for 10 days. Plant total fresh weight and morphology were analyzed. Root hair formation was evaluated by counting root hairs in the terminal zone (1.5 cm) of the primary root. Lateral root formation was analyzed by counting lateral roots (of 2 mm minimum length) in the whole root system. Primary root length was measured using the program ImageJ version 1.53. Images were taken with a digital camera (EOS M50, Canon, USA). The experiment was repeated 3 times.

#### *A. thaliana* Growth Conditions for SCA7 Rhizosphere Competence

Sterilized *A. thaliana* seeds were grown for 7 days on $\frac{1}{2}$ MS agar plates. SCA7 cultures were pre-grown in NB. Overnight-grown cultures were washed two times in 1 x PBS and adjusted to 2 × 10^7^ CFU/ml. Seedlings were incubated for 1 h in SCA7 bacterial solution or in 1 x PBS as mock treatment at 30°C. Inoculated seedlings were planted in sterile Phytatray II (Sigma) boxes filled with 80 ml sterilized quartz sand and 20 ml Hoagland's solution (Sigma), and sealed with Parafilm. Boxes were placed for 2 weeks in a growth chamber (Weiss Technik, Modell SGC120PG2) at 23°C, 55% humidity, and a PAR of approximately 80 μmol photons m^−2^ s^−1^, with a 12 h light−12 h dark interval. Subsequently, roots were harvested with sterile forceps, washed in 1 x PBS, fixed in 55% (v/v) EtOH and 1 x PBS mix, and stored at −20°C until further use.

#### Fluorescence *in situ* Hybridization and Confocal Laser Scanning Microscopy

Fluorescence *in situ* hybridization (FISH) was performed according to Alquéres et al. (2013), with modifications as described in Kuhl et al. (2021), using chemicals from AppliChem (Darmstadt, Germany). Briefly, the roots were desiccated in an increasing ethanol series (50%, 80%, 96%) for 3 min each. Afterward, the roots were submerged in a hybridization buffer, containing 15 pmol of the fluorescently labeled probes EUB338, specific for bacteria (Amann et al., 1990; Daims et al., 1999) and labeled with fluorescein [FITC (488 nm), Thermo Scientific, Bremen, Germany], and Gam42a (Manz et al., 1992), specific for gamma-proteobacteria labeled with Cy3 (561 nm, Thermo Scientific). Hybridization was performed for 1.5 h at 46°C. Bacterial cells on the roots were investigated using confocal laser scanning microscopy (CLSM) at the Zeiss LSM 880 (Zeiss, Jena, Germany), equipped with an argon ion laser and a helium neon laser for excitation of FITC (488 nm), Cy3 (561 nm), and an unlabeled control channel (633 nm). Bacterial cells were observed with a C-Apochromat 63x/1.20 W Korr M27 water immersion objective. Micrographs were taken using Zeiss software Zen Black Edition 2.3 SP1 FP1 (Version 14.0.12.201, Zeiss).

#### SCA7 Growth-Promoting Activity on *T. aestivum* Plants

SCA7 cultures were pre-grown in NB. Overnight-grown cultures were washed two times in 1 x PBS and adjusted to 2 x 10^7^ CFU/ml. Sterile *T. aestivum* seedlings with equal shoot and root size were washed two times in sterile water to remove potential antibiotic residues. Subsequently, they were incubated at 30°C for 1 h in the bacterial solution or in 1 x PBS as mock treatment. Dead bacteria were not included, because results of the previous experiment showed that the plant did not respond differently on the dead bacteria compared to the 1 x PBS control. A total of 15 seedlings of each treatment were planted in pots filled with 90% quartz and 10% sand as well as 20 ml Hoagland's solution. Pots were placed randomly in a growth chamber (Weiss Technik, Modell SGC120PG2) at 23°C, 55% humidity, and approximately 80 μmol m^−2^ s^−1^ light intensity, with a 12 h light−12 h dark interval. The pots were watered every second day with 40 ml water and additional 20 ml Hoagland's solution after 2 weeks. After 3 weeks, shoot and root fresh and dry weight, as well as root and shoot length, was evaluated. The experiment was repeated two times.

#### *Pst* DC3000 Infection Assays on *A. thaliana* in Presence of SCA7

Stratified Col-0 seeds were sown onto soil (Pikiererde, Classic Profisubstrat, Einheitserde) and placed in a growth chamber for 7 days. Subsequently, the seedlings were separated into pots and grown for additional 3 weeks. An overnight culture of SCA7 in a volume of 200 ml was centrifuged at 2,400 x *g* for 10 min, and the pellet was suspended in sterile water. Two dilutions of 2 x 10^8^ CFU/ml and 4 x 10^7^ CFU/ml were prepared to determine the most effective cell concentration. Three-week-old plants were inoculated by syringe with 1 ml of SCA7 from each dilution or with sterile PBS (mock) at the soil surface near the roots. After 24 h, the plants were infected with *Pst* DC3000 by spraying a 1 x 10^8^ CFU/ml in 10 mM MgCl_2_, with 0.4% (v/v) μl Silwet L-77 suspension according to Katagiri et al. (2002) (Supplementary Figure S1). After 1 h, the plants in trays with lids were put back into the growth chamber. Leaves were harvested 3 days after infection with *Pst* DC3000. Two same-age leaves of two plants were treated as one sample. Sample material was weighed with a fine scale, and then 10 mM MgCl2 was added (400 μl), and material was grounded with a pestle. Dilutions of 10^−2^, 10^−3^, and 10^−4^ were prepared, and 10 μL per dilution was plated onto NYG agar plates, supplemented with rifampicin (50 μg/L) and kanamycin (30 μg/L) and turned in vertical direction to let the drop run across the plate. The plates were incubated at 30°C for 3 days, and the number of antibiotic-resistant CFU representing *Pst* DC3000 cells was determined. The number of CFU was normalized to the fresh weight of leaves (CFU/mg of FW), indicating the pathogen infestation of the plants.

#### Sampling of Plant Material and RNA Extraction for qRT-PCR Analyses

For gene expression analyses in leaves after treatment, leaves treated with 1 x PBS or SCA7 alone or in combination with the pathogen *Pst* DC3000 samples were harvested at 24 h after infection with SCA7 or *Pst* DC3000, respectively (Supplementary Figure S1). The time point was chosen according to De Vos et al. (2005). A sample consisted of 3 middle-aged leaves harvested from 3 different plants. Samples were frozen in liquid nitrogen immediately after harvesting and stored at −80°C.

For gene expression analyses in roots after treatment with SCA7, surface-sterilized and stratified *A. thaliana* seeds were sown on MS plates (120 cm × 120 cm) and placed in the growth chamber for 7 days. Twelve seedlings were preselected and transferred to new MS petri dishes for another week of growth. Plant roots were inoculated with 10 μL of SCA7 suspension (2 x 10^7^ CFU/ml) or 1 × PBS (mock). Roots were cut off with a scalpel and harvested at 24 h after the treatment. The samples containing 12 plant roots each were immediately frozen in liquid nitrogen and stored at −80°C until RNA extraction.

Total RNA was obtained according to the manufacturer's instructions of the RNeasy^®^ Plant Mini Kit (QIAGEN GmbH, Hilden, Germany) used for extraction. Approximately, 200 ng of RNA per sample was used for cDNA synthesis with Oligo [dt]12–18 (500 μg/ml) and M-MLV Reverse Transcriptase (Invitrogen, Thermo Fisher Scientific) in a total volume of 20 μL. Quantitative real time (qRT)-PCR was performed for the genes listed in Supplementary Table S1 using the Sso Advanced TM Universal SYBR^®^ Green Supermix (Bio-RAD, Feldkirchen, Germany). All the experiments were performed with three technical replicates. qRT-PCR conditions: 95°C for 3 min, followed by 40 cycles of 95°C for 10 s and 60°C for 30 s, ending with 95°C for 10 s and a melt cycle from 65 to 95°C. Expression of the genes of interest was normalized to the expressions of the housekeeping genes *AtEF1*α and *AtACTIN7*. Sequences of all primers and their references are listed in the appendix (Supplementary Table S1). Calculations of expression values were performed with the software CFX Maestro (Version 2.0; Bio-RAD).

### Statistical Analysis

Statistical analysis was performed with RStudio 3.6.1 using the packages “sciplot” and “ggplot2,” where data were tested for normal distribution with the Shapiro–Wilk test and analyzed with Students' T-test, ANOVA or non-parametric tests, followed by *post hoc* analyses. The significance level of 5% was marked by asterisks in the graphs. Sample size was not predetermined using statistical methods.

## Results

### Genomic Analyses of SCA7

#### Genome Properties of SCA7

Genomic DNA of SCA7 was sequenced using the PacBio^®^ long-read sequencing technology. Subsequently, the SCA7 genome was assembled from 579,331 total subreads, with an average (N50) seed read length of 10,415 bp and 284 x coverage, using the Microbial assembly pipeline of the single molecule real-time (SMRT) portal interface (v9.0 PacBio SMRTLink^®^, Pacific Biosciences), with default parameters and an internal quality check. The sequence assembly produced one not-circular closed contig, with 6,782,730 bp and 59.1% G+C content available at NCBI accession number NZ_CP073104 (Supplementary Table S2).

#### SCA7 Phylogeny

In order to identify the isolate SCA7 and to assign a phylogenetic taxonomy to it, we performed a phylogenetic analysis based on the 16S rRNA gene sequence and the whole genome sequence. According to the 16S rRNA gene sequence analysis, SCA7 is most closely related to the type strains of *Pseudomonas reinekei* (99.6%), followed by *Pseudomonas helmanticensis* (99.2%), *Pseudomonas baetica* (99.1%), *Pseudomonas koreensis* (99.2%), and *Pseudomonas jessenii* (99.4%). The most closely related type strains based on digital DNA-DNA-Hybridization (dDDH) values of whole genome sequence comparison using Type Strain Genome Server (TYGS) was *Pseudomonas helmanticiensis* BIGb0525, with a dDDH value of 74.3%, followed by eleven other *Pseudomonas* strains (Table 1), with dDDH values below the species delineation threshold of 70%.

**Table 1:** Digital DNA-DNA-Hybridization values of related strains to SCA7 generated with the Type Strain Genome Server (TYGS) (Meier-Kolthoff and Göker, 2019).

**Type strains closely related to SCA7**	**dDDH (in %)**
*Pseudomonas helmanticiensis* BIGb0525	74.3
*Pseudomonas baetica* LMG 25716	54
*Pseudomonas atagonensis* PS14	60.2
*Pseudomonas koreensis* LMG 21318	59.2
*Pseudomonas koreensis* JCM 14769	58.9
*Pseudomonas granadensis* LMG 27940	58.3
*Pseudomonas atacamensis* M7D1	54
*Pseudomonas moraviensis* LMG 24280	57.3
*Pseudomonas glycinae* MS586	53.9
*Pseudomonas kribbensis* KCTC 32541T	51.9
*Pseudomonas reinekei* MT1	35.7
*Pseudomonas jessenii* DSM 17150	35.8
*Pseudomonas prosekii* LMG 26867	41.1

**Figure 1:**
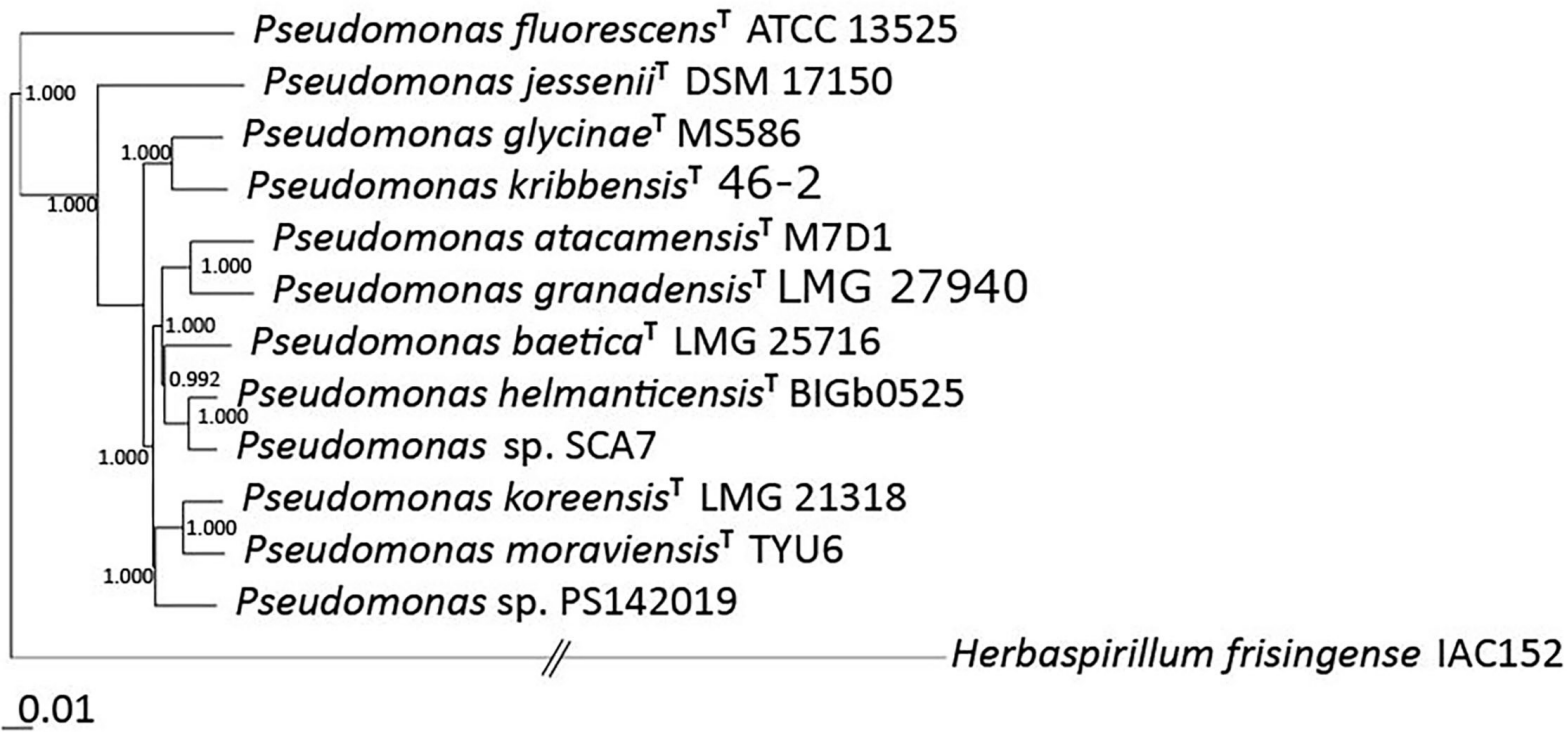
The maximum-likelihood phylogenetic tree of *Pseudomonas* sp. SCA7 and 11 closely related type strains generated with FastTree 2.1 from 1,510 genes of the core genome. Values represent local support values based on the Shimodaira–Hasegawa test (1 SH = 100% bootstrap). The scale bar represents nucleotide substitutions per site (0.01 scale = 1% nucleotide substitutions per site).

The phylogenetic tree for 307 *Pseudomonas* genomes was built out of 70 core genes of the genomes from 21,630 in total, using the algorithm implemented in EDGAR 3.0 (Blom et al., 2016) (Supplementary Figure S4). The phylogenetic subtree for 12 *Pseudomonas* genomes was built out of 1,510 core genes of the genomes from 19,630 in total, using the algorithm implemented in EDGAR 3.0 (Blom et al., 2016) (Figure 1) and revealed a close relation to *P. helmanticensis*. The average nucleotide identity (ANI) matrix based on 11 most closely related *Pseudomonas* strains revealed ANI values of 96.23% with *Pseudomonas koreensis* CFBP13504 (NZ_QFZV01000085) and 97.23% with *Pseudomonas koreensis* CI12 (NZ_MPLD01000016) (Supplementary Figure S3), which are ANI values above the species delineation threshold of 94%. The ANI matrix based on 11 most closely related *Pseudomonas* type strains revealed closest relation to *Pseudomonas helmanticensis* (92.04%), but all ANI values in comparison of the type strains were below the species delineation threshold of 94% (Supplementary Figure S2). Taken together, the 16S rRNA gene-based and whole genome-based analyses with dDDH and ANI values suggest that SCA7 cannot be unambiguously assigned to *P. helmanticensis*.

#### SCA7 Genome Annotation

To predict potential traits of SCA7 that may guide the experimental functional characterization, the genome was analyzed using several annotation tools. For the genome of SCA7, a total of 5,981 and 5,982 coding sequences were predicted with PGAP and RAST, respectively, with 97% annotation agreement and 52% of the coding sequences were sorted in 26 main RAST subsystems and 557 subsystems (categories). RAST identified genes known to function in volatile metabolism and synthesis, production of antifungal and antibacterial compounds, antibiotic resistance, flagella biosynthesis and motility, auxin biosynthesis, siderophore receptors, and biosynthesis, as well as the channel protein secretin and the effector protein HopPmaJ (Supplementary Table S4). The identified genes were also detected in PGAP (Supplementary Table S4). Furthermore, the tool CARD predicted seven antibiotic resistance gene homologs (Supplementary Table S5), the tool VFDB identified 136 virulence factors (VFs) (Supplementary Table S6), and the tool antiSMASH revealed 11 biosynthetic gene clusters (BGC), with the potential to produce antifungal compounds, arylpolyene, which is a yellow pigment protecting against reactive oxygen species (ROS), osmotic stress-protecting compounds, antibiotics or siderophores (Supplementary Table S3). Apart from the lokisin BGC with the highest similarity of 85%, all other BGCs have a lower similarity up to 40%. Identification of these genes indicated that SCA7 has the potential to display plant growth-promoting and biocontrol activities.

### Colonization and Plant Growth-Promoting Abilities of SCA7

The isolation of SCA7 from roots of *T. aestivum* indicates a close association with plants, and the genome annotation revealed the potential of SCA7 to show plant growth-promoting activities, which were further analyzed to elucidate functionality of the identified traits.

#### SCA7 Can Swarm and Produce Biofilm *in vitro*

**Figure 2:**
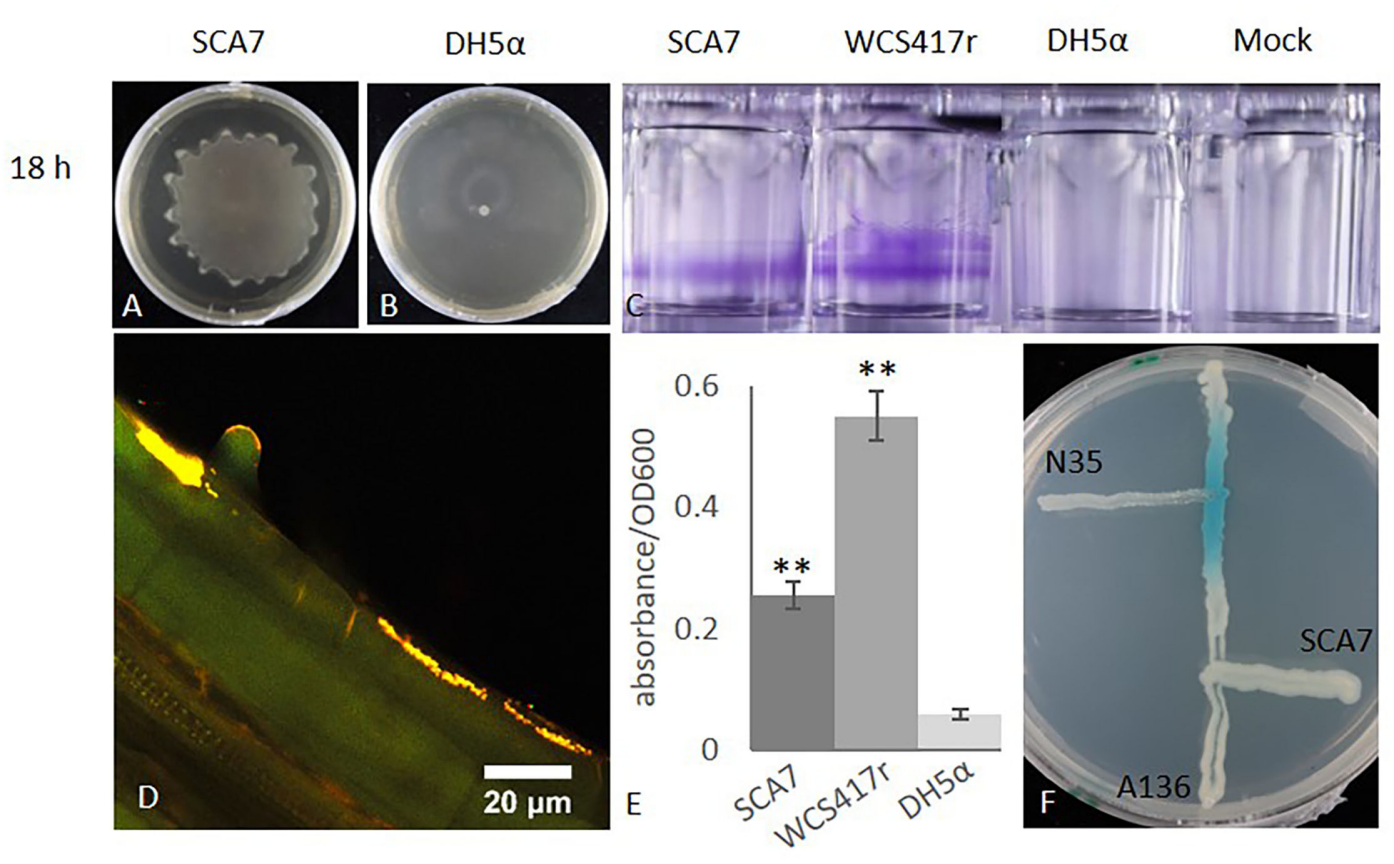
Rhizosphere competence of SCA7. Swarming ability of SCA7 **(A)** and DH5α **(B)** on a semi-solid medium after 18 h. Ability to produce biofilm *in vitro* **(C)** and quantification of biofilm production compared to WCS417 (positive control) and DH5α (negative control), with averaged results of 12 replicates normalized to OD600 = 1 **(E)**. Absorbance measured at 550 nm; error bars indicate standard deviation, **p = 0.001677. **(D)** *In situ* detection of root colonization of *Arabidopsis thaliana* roots by SCA7 on 2-week-old *Arabidopsis thaliana* roots grown in sterile quartz sand and visualized after fluorescence *in situ* hybridization (FISH) in a confocal laser scanning microscope (CLSM). Bacterial cells are identified by yellow color from overlayed channels of probes EUB (green) and gam42 (red). **(F)** An AHL biosensor assay with biosensor strain A136, positive control N35, and SCA7. Blue color indicates AHL production.

Swarming motility and biofilm formation are important traits of (plant beneficial) rhizobacteria because they facilitate to effectively colonize the plant roots. The genomic analysis indicated presence of genes involved in biosynthesis and motility of the flagella (Supplementary Table S3, S6), and SCA7 displayed a swarming zone around the initial inoculation spot after 18 h of incubation that increased its radius with progressing time (Figure 2A), whereas the negative control *E. coli* DH5α did not (Figure 2B). Similar to the positive control, *P. simiae* WCS417r, SCA7 was able to produce a biofilm on the walls of inoculated wells indicated by formation of a violet ring after crystal violet staining, whereas the negative control and mock treatment could not (Figure 2C). Moreover, quantification of produced biofilm revealed that SCA7 and *P. simiae* WCS417r showed a significantly higher level of biofilm formation compared to the negative control *E. coli* DH5α, (Fligner–Killeen test, *p* = 0.001677, Figure 2E). In detail, SCA7 showed a 4-fold increase in biofilm formation compared to *E. coli* DH5α and 0.5-fold less biofilm formation than the positive control *P. simiae* WCS417r. Interestingly, genes involved in quorum sensing by AHL production could not be unambiguously identified (Supplementary Table S6), and the AHL biosensor assay with SCA7 was negatively compared to the positive control *A. radicis* N35 (Figure 2F). Nevertheless, genes encoding for transcriptional regulator of the LuxR family, the enzyme acyl-homoserine lactone acylase (Supplementary Table S4), which is able to degrade AHLs, as well as the two component regulatory system GacA/GacS, were identified in the SCA7 genome, which could be involved in sensing and signaling relevant to biofilm production. These experiments revealed that SCA7 possesses the ability of swarming motility and biofilm formation under static batch-growth conditions, which can facilitate adherence to the roots and, therefore, indicates the potential of SCA7 to successfully colonize plant roots. However, the biofilm formation is probably not mediated by the classical AHL quorum-sensing system.

#### SCA7 Colonizes *A. thaliana* Root (Epidermal Cells)

In order to investigate if SCA7 actually colonizes plant roots, *A. thaliana* plants were co-cultivated with SCA7 for 2 weeks in an axenic system, and harvested roots were subsequently analyzed using FISH and CLSM. Bacteria were detected on roots inoculated with SCA7. All localized bacteria were labeled by both probes EUBI-III and Gam24a, as revealed by the yellow color of overlapping channels (Figure 2D), confirming their identity as SCA7 cells in the axenic system. Mock-treated plants did not carry any bacterial cells and exhibited the general auto fluorescence of plant material (data not shown). SCA7 colonization occurred at the root surface of mature root parts and at the base of emerging lateral roots. Agglomerations were especially detected around the base of root hairs, as well as on the root hair surface and tip. Occurrence of SCA7-labeled bacteria decreased toward the primary root tip, with no visible agglomerations at the tip itself or adjacent border cells (data not shown). Endophytic colonization was not observed during the experimental period. Thus, SCA7 is able to colonize the epidermal root layers of *A. thaliana*, mainly at the root hair zone.

#### SCA7 Produces IAA and Siderophores *in vitro*

**Figure 3:**
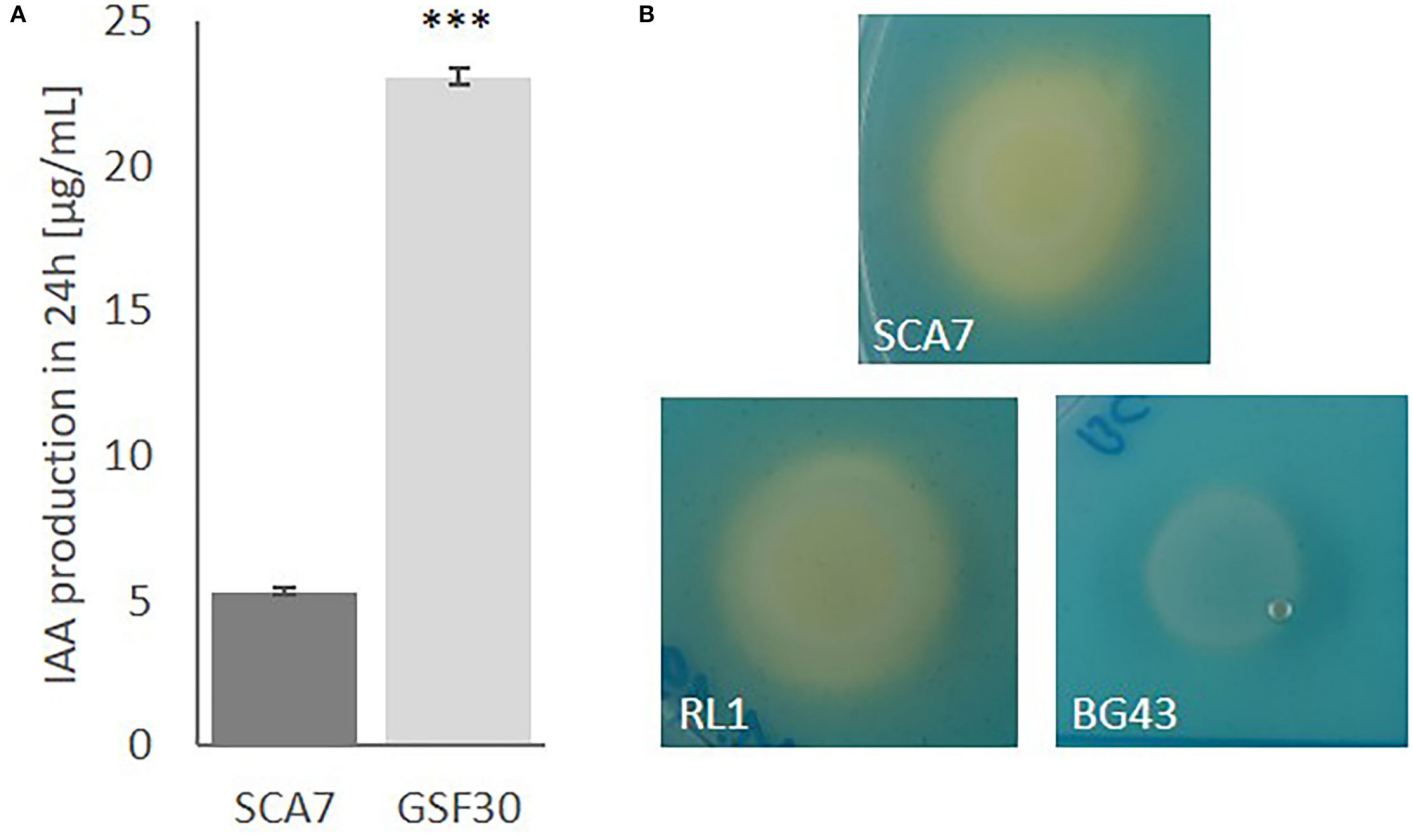
Plant growth-promoting traits of SCA7 *in vitro*. **(A)** IAA production after 24 h normalized to OD_600_ = 1 with *Herbaspirillum frisingense* GSF30 as positive control. Error bars indicate standard deviation, *N* = 3, ***p = 0.00000012 **(B)** Siderophore production indicated by color change of the medium from blue to orange with RL1 as positive control and BG43 as negative control.

Our genome analysis suggested that SCA7 harbors common plant growth-promoting traits, which were analyzed *in vitro* to see if the genes involved in auxin and siderophore production identified in the genome annotation were actually transferred into functional traits. After 24 h, SCA7 was able to produce 5.3 μg/ml of IAA on average, although less compared to the positive control *H. frisingense* GSF30, which produced 23 μg/ml on average (Figure 3A). Furthermore, the analysis of siderophore production revealed that SCA7 has as well the ability to produce siderophores, indicated by a color change of the overlay agar from blue to orange (Figure 3B). These results showed the ability of SCA7 to produce IAA and siderophores, indicating functionality of the genetically identified traits.

#### SCA7 Modifies the Root Architecture and Promotes Growth in *A. thaliana* and *T. aestivum*

**Figure 4:**
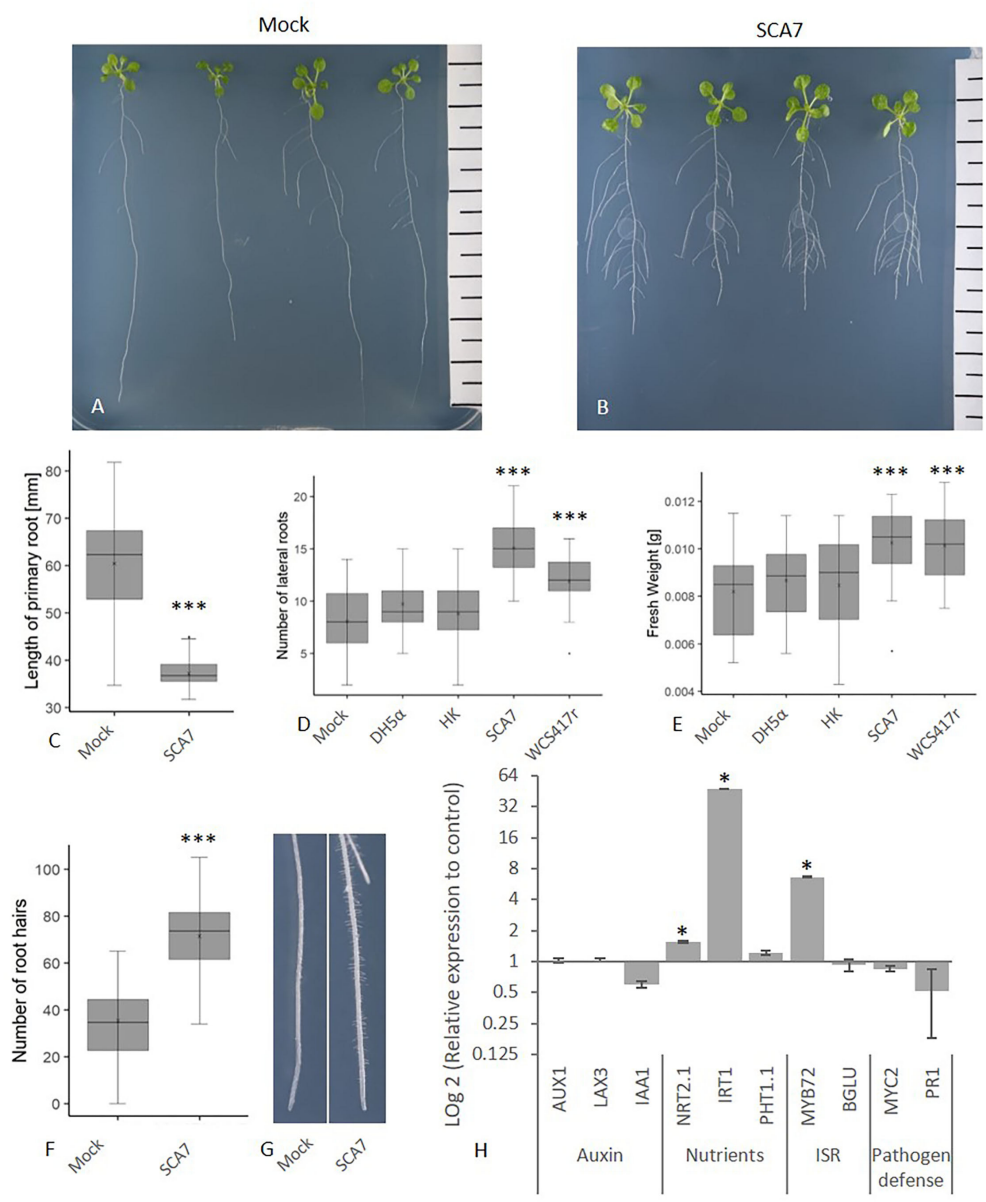
Ability of SCA7 to change root architecture and promote plant growth in *Arabidopsis thaliana* seedlings. Representative seedlings after 10-day growth on ½ MS agar **(A)** without and **(B)** with SCA7. **(C)** Primary root length difference of seedlings in absence or presence of SCA7. Number of **(D)** lateral roots and **(E)** fresh weight of seedlings treated with SCA7. Differential root hair formation on the terminal 1.5 cm of the primary root in absence or presence of SCA7 **(F,G)**. Results averaged from three experiments each with 10 replicates. **(H)** Relative to control expression of marker genes after 24 h involved in auxin, nutrient uptake, and defense responses in *A. thaliana* seedling roots inoculated with SCA7. Mock represents control treatment with PBS, WCS417r (positive control), HK (heat-killed SCA7), and DH5α as negative control. Results were obtained from three pooled experiments. Asterisks indicate significant differences (*p* value * < 0.05, ** < 0.01, *** < 0.001). Error bars indicate standard deviation.

**Figure 5:**
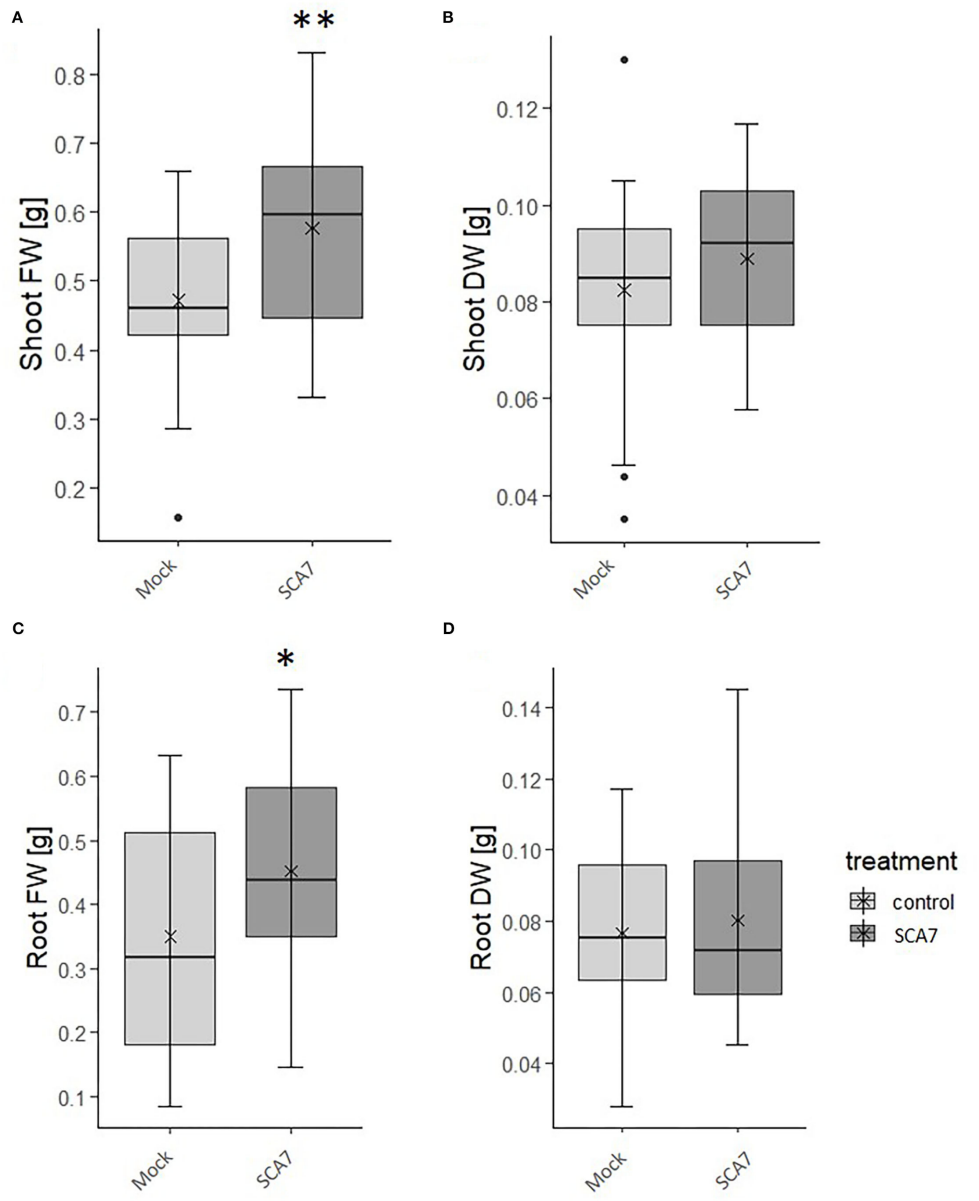
Influence of SCA7 on **(A,C)** fresh weight and **(B,D)** dry weight in **(A,B)** shoots and **(C,D)** roots in *Triticum aestivum* grown in sand-clay system for 3 weeks. Results averaged from two experiments each with 15 replicates. Asterisks indicate significant differences (*p* value* < 0.05, ** < 0.01). DW = dry weight, FW = fresh weight. Error bars indicate standard deviation.

To confirm the results of the *in vitro* experiments for plant beneficial traits, the plant growth-promoting potential of SCA7 was subsequently analyzed on the reference dicot *A. thaliana* and the monocot *T. aestivum*, the original host of SCA7 and one of the most important crops worldwide. *A. thaliana* plants inoculated with SCA7 exhibited a significantly shorter primary root length (ANOVA, p = 3.27e^−13^) (Figures 4A–C) and a significantly higher number of lateral roots (ANOVA, *p* = 2e^−16^) (Figure 4D), which even exceeded the numbers of the positive control WCS417r. Moreover, SCA7-treated plants displayed an increased complete fresh weight of the plants (ANOVA, p-value = 3.88e^−7^) (Figure 4E), as well as an increased number of root hairs on the terminal 1.5 cm of the primary root (Figures 4F,G). Negative controls of heat-killed bacteria, *E. coli* DH5α, and mock treatment induced no changes in root architecture, lateral root and root hair formation, and fresh weight in *A. thaliana*. *T. aestivum* plants inoculated with SCA7 grown in the sand-clay system (Figures 5A,C) had a significantly increased root (*t*-test; *p* = 0.0257) and shoot (*t*-test; *p* = 0.0015) fresh weight by 29.2% and 22.4%, respectively, compared to mock treatment. Dry weight showed no significant difference between the treatments (Figures 5B,D). *T. aestivum* root architecture could not be characterized because of the massive branching in this system. Taken together, these results indicate that SCA7 has the ability to modulate the root architecture (increase of lateral roots and of root hairs) and to increase the fresh weight of *A. thaliana* plants in axenic conditions and the fresh weight of *T. aestivum* in the sand-clay system. The effects of SCA7 on *A. thaliana* are thus comparable to the well-characterized *P. simiae* WCS417r (Pieterse et al., 2020).

#### SCA7 Alters Expression of Genes Involved in Iron Deficiency Response and ISR in *A. thaliana*

In order to investigate the early effect of SCA7 on the plant gene expression in root tissue, the transcript levels of several marker genes for auxin uptake and signaling, nutrient uptake, and defense responses were analyzed (Figure 4H). Twenty-four h after inoculating roots with the SCA7 strain, the relative expressions of *AUX1* (an auxin influx transporter) and *LAX3* (an auxin influx carrier), both involved in auxin polar transport and lateral root formation, were not different from roots inoculated with a phosphate saline buffer (PBS, control). *IAA1* (induced by auxin) expression was slightly downregulated in roots treated with SCA7 compared to control. This indicated that, at 24 h of application of SCA7, auxin-related responses in root cells were not activated under the tested conditions. Relative expression of *NRT2.1* (a nitrate transporter) and of *IRT1* (an Fe^2+^ transporter) was significantly upregulated, whereas expression of *PHT1;1* (an inorganic phosphate transporter) remained basically unchanged, compared to control. Regarding ISR, *MYB72* (a transcription factor mediator of ISR) appeared significantly upregulated after SCA7 treatment compared to control, whereas expression of *BGL42*, a downstream *MYB72*-target, was not affected by SCA7. Interestingly, the pathogenesis-related marker gene *PR1* was slightly downregulated, as well as the transcription factor *MYC2* in roots treated with SCA7 compared to the mock control. This indicates that the usual defense responses upregulated by pathogenic *Pseudomonas*, such as *Pst* DC3000, are not mounted by plants when in contact with SCA7 at early time points (24 h). These preliminary results provide a first insight into the effect of SCA7 on roots. Iron deficiency responses appear clearly activated, resulting in upregulation of iron and nitrate transport, together with the upregulation of the MYB72 transcription factor.

### Interaction With Other Microorganisms

#### SCA7 Restricts the Growth of Specific Fungi and Bacteria in Confrontation Assays

**Figure 6:**
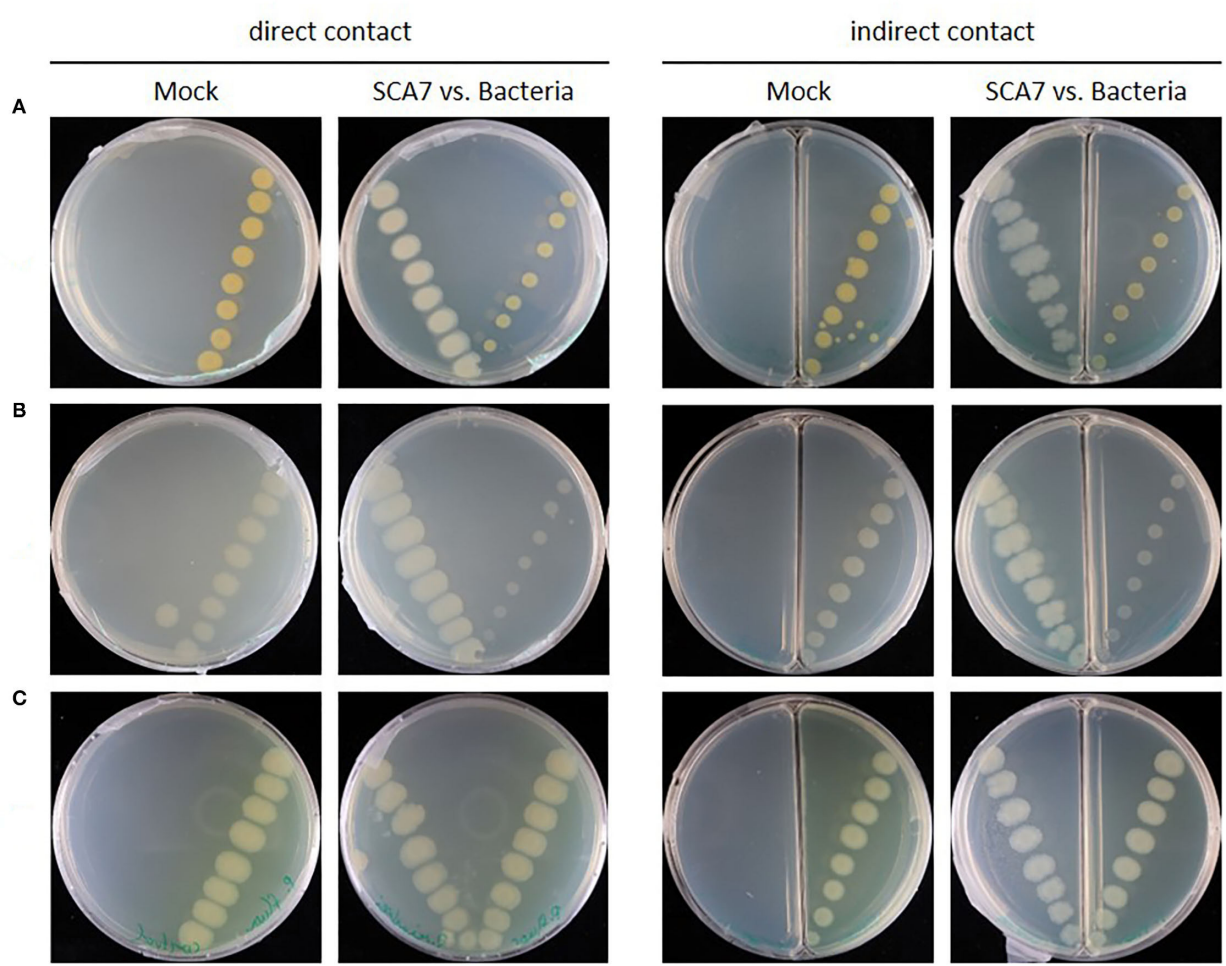
A confrontation assay of SCA7 against plant pathogenic bacteria **(A)** *Xanthomonas translucens*, **(B)** *Pseudomonas syringae pv. tomato DC3000* and plant beneficial, and **(C)** Bacillus velezensis FZB42 in direct (left) and indirect (right) contact. Antagonistic activity is indicated by smaller bacterial colonies. Control plates (= **Mock**) without SCA7.

**Figure 7:**
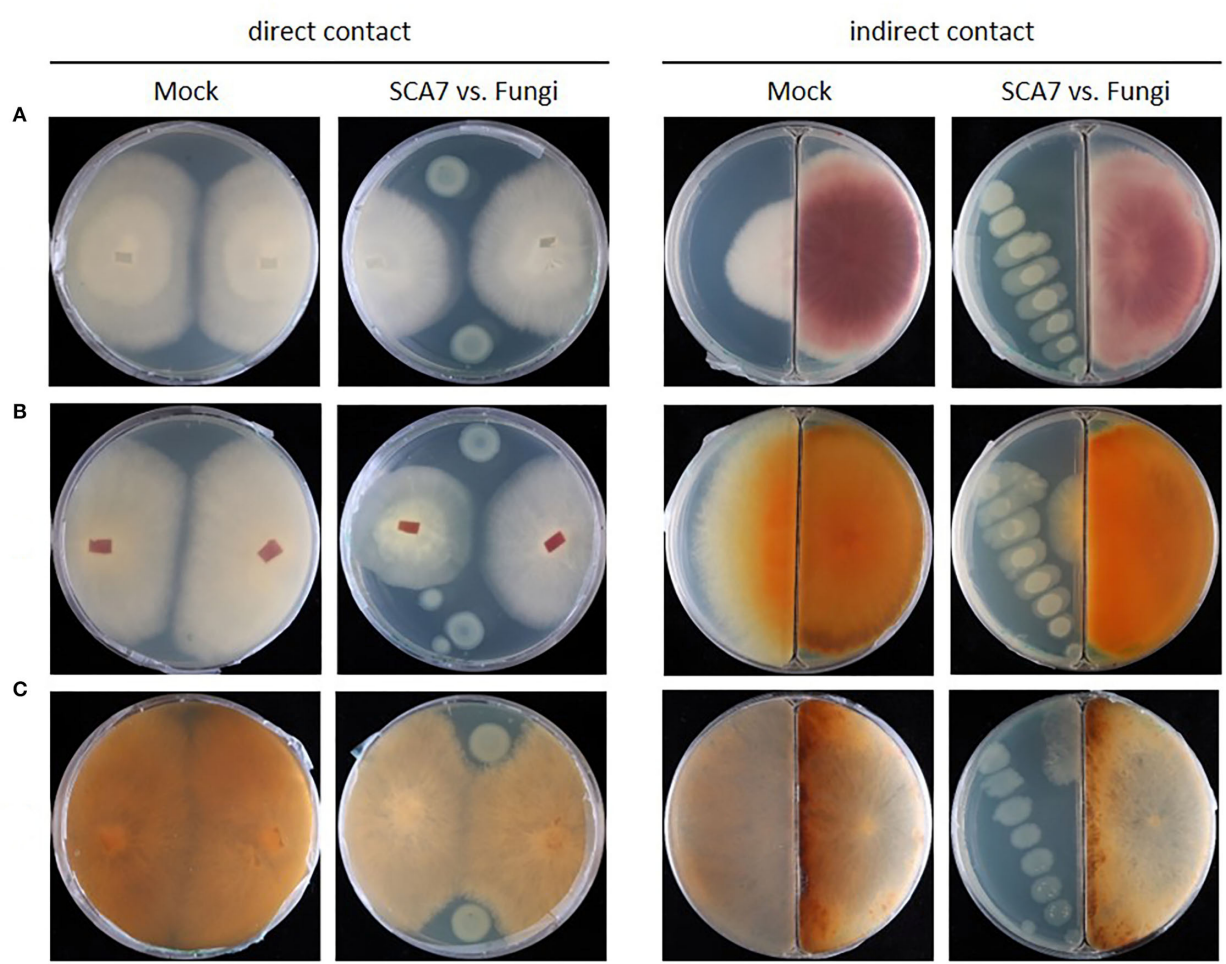
A confrontation assay of SCA7 against plant pathogenic fungi **(A)** *Fusarium oxysporum*, **(B)** *Fusarium culmorum*, and **(C)** *Rhizoctonia solani* in direct (left) and indirect (right) contact grown for 7 days. Antagonistic activity is indicated by an inhibition zone around bacteria. Control plates (= **Mock**) without bacteria.

Gene clusters potentially involved in the production of antimicrobial compounds were identified in the SCA7 genome, which can become relevant to antagonistic effects against pathogens. Therefore, SCA7 was tested for its antagonistic activity against the plant-pathogenic fungi *Fusarium oxysporum, Fusarium culmorum*, and *Rhizoctonia solani* (Figure 6) and the plant-pathogenic bacteria *X. translucens* and *Pst DC3000* (Figure 7) in direct as well as indirect contact. In direct contact with SCA7, *F. oxysporum* and *F. culmorum* showed a reduced growth radius compared to mock treatment, whereas *R. solani* expanded across the whole plate, with a delimited growth inhibition zone around the SCA7 colony. In the split plate assay, substantially less mycelia of all tested fungi were observed to grow over from the side of fungi to the side with SCA7 bacterial colony compared to fungi grown in plates without SCA7. SCA7 reduced also the growth of the plant-pathogenic strains *X. translucens* and *Pst DC3000* in direct and indirect contact (Figure 7). In direct confrontation, pathogen growth was reduced with decreasing distance to SCA7. Growth of the plant beneficial strain *B. velezensis* FZB42 was not inhibited by SCA7 (Figure 7C). These experiments demonstrate that SCA7 displays an ability to restrict and inhibit the growth of plant pathogenic fungi and bacteria in direct and indirect contact.

#### The Major mVOC Released by SCA7 Is 1-Undecene

**Figure 8:**
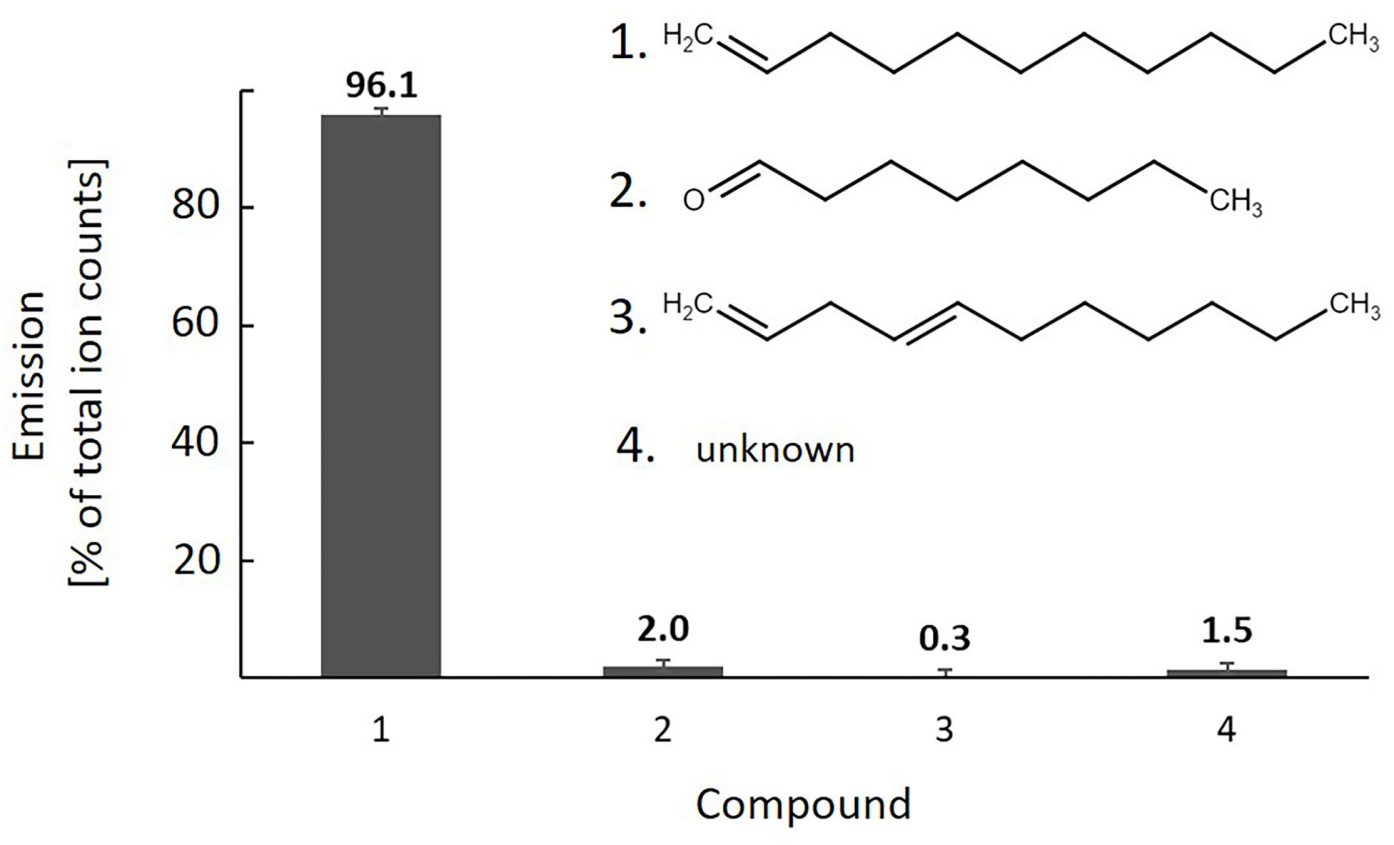
mVOCs detected in the headspace of *Pseudomonas* sp. SCA 7 cultures. The emission is reported as percentage (mean ± SD) of total emission for (1) 1-undecene, (2) octanal, (3) 1,4-undecadiene, and (4) an unknown compound. The chemical structures of the compounds 1 to 3 are given within the graph (drawn with Marvin JS by ChemAxon LtD; http://www.chemaxon.com). Further characteristics of the detected compounds, including retention time, Kovats retention index, and Chemical Abstracts Service (CAS)–registry numbers, are given in the Supplementary Table S7.

As SCA7 was able to reduce the growth of plant pathogens in a shared headspace without physical contact, we wondered if the ability could be mediated by the production of mVOCs. The GC-MS analyses revealed emission of four compounds by the pure SCA7 cultures grown in NB media. The most prominent mVOC detected in the emission pattern of SCA7 was the alkene 1-undecene, accounting for 96% of the total emission (Figure 8). The other detected mVOCs were putatively annotated as octanal (2.% of the total emission) and 1,4-undecadiene (0.3% of the total emission), whereas the 4th detected compound (1.5% of the total emission; Kovats retention index: 1,653) remained unidentified (Figure 8; Supplementary Table S7). The SCA7 genome harbors genes encoding enzymes for the biosynthesis of the compounds 1-undecene and 2,3-butanediol, which are involved in plant growth promotion and biocontrol mechanisms. The analysis indicated the production of the identified mVOCs by SCA7 in unchallenged conditions, which could play a role in the observed plant growth-promoting and biocontrol effects.

#### SCA7 Confers Resistance to *A. thaliana* Against Infection With Pst DC3000

**Figure 9:**
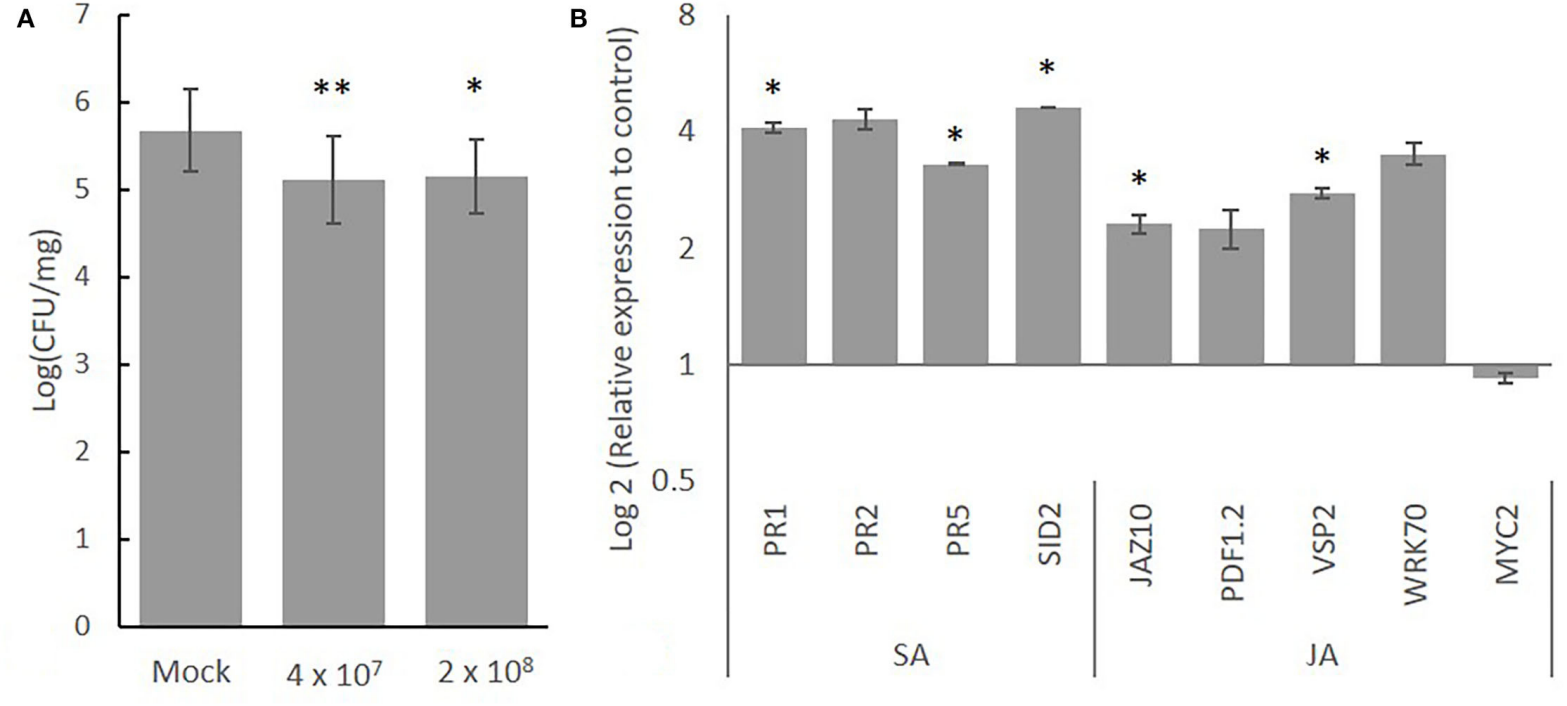
Effect of SCA7 on *Pst* DC3000 infection in *A. thaliana*. **(A)** *Pst* DC3000 proliferation (CFU/mg fresh weight) in *A. thaliana* leaf tissue. Plant roots were inoculated with SCA7 with 4-x-10^7^ CFU/ml and 2-x-10^8^ CFU/ml. Results averaged from three experiments (N = 4, N = 8, N = 8). Asterisks indicate significant differences (*p* value * < 0.05, ** < 0.01). **(B)** Effect of SCA7 on the relative expression of genes involved in common induced defense responses in *A. thaliana* shoots infected with *Pst* DC3000 and co-inoculated with SCA7 (or PBS, as control) at the roots. Three-week-old plants were inoculated with SCA7 and PBS at the roots, and then, after 24 h, the plants were spray infected with a suspension of *Pst* DC3000. Leaves were harvested after 24 h of infection with the pathogen, and expression of marker genes involved in defense responses mediated by SA and JA and was profiled. Error bars indicate standard deviation.

In order to evaluate if the observed antagonistic effect of SCA7 against plant pathogenic microbes can actually protect plants, the antagonistic activity of SCA7 was tested in the well-known plant-pathogen system of *A. thaliana* and *Pst* DC3000. Therefore, roots of *A. thaliana* plants were first inoculated with SCA7, and, later, leaves of the same plant were infected with *Pst* DC3000. *A. thaliana* plants inoculated with the strain SCA7 at the roots resulted to be more resistant to spray infection with *Pst* DC3000, shown by the significantly (ANOVA, *p* = 0.000478) lower numbers of pathogenic bacteria on plants inoculated with SCA7 compared to plants inoculated with 1 x PBS (Figure 9A). Two different SCA7 concentrations were tested, and both treatments showed increased resistance toward *Pst* DC3000, with no significant (ANOVA, *p* = 0.94) difference among them. These results revealed that independent from the applied inoculation doses, the presence of SCA7 on soil-grown *A. thaliana* roots induced a growth restriction effect against the pathogen *Pst* DC3000 attacking plant leaves. The antagonistic activity of SCA7 against this specific pathogen observed previously in *in vitro* experiments was, therefore, also confirmed *in planta*.

#### SCA7 Treatment Upregulates SA- and JA/ET-Related Gene Expression in *A. thaliana* During Infection With *Pst* DC3000

The antagonistic effect of the root-inoculated SCA7 against the leaf-pathogen *Pst* DC3000 was observed, although the bacteria were applied distantly. The effect could be mediated by the identified mVOCs and by the ability to prime plants against pathogens by altering defense-related signaling pathways in plants. Therefore, the expression patterns of defense-related genes of *A. thaliana* plants inoculated with SCA7 or mock treated were analyzed after infection with *Pst* DC3000. The expression of all four tested SAR-related marker genes, i.e., *PR1, PR2, PR5*, and *SID2*, was upregulated in shoots at 48 h after root treatment with SCA7 and 24 h after leaf infection with the pathogenic *Pst* DC3000 (Supplementary Figure S1), compared to control plants treated with PBS and, later, infected with *Pst* DC3000 (Figure 9B). Similarly, relative expression of JA-signaling marker genes, *JAZ10, VSP2, WRKY70*, and JA/ET-signaling marker *PDF1.2* was upregulated at 48 h after root treatment with SCA7 and 24 h after leaf infection with the pathogenic *Pst* DC3000 compared to the corresponding control treatment (Figure 9B). To know if these responses—usually activated during defense—are also regulated by SCA7 alone in leaf tissues without pathogen leaf infection, the expression of chosen genes was profiled in leaves from plants inoculated only with SCA7 or with PBS (as control) after 24 h. Here, the expression of *JAZ10, VSP2*, and *SID2* was actually downregulated (Supplementary Figure S5). Relative gene expression of *PR1* and *PR2* was unmodified, and *PR5* relative expression was slightly upregulated. These results suggest that SCA7 activates or primes generic plant defense responses dependent on SA or JA simultaneously when under attack of pathogens (in our case, a pathogenic bacterium), as these responses are not activated by SCA7 alone.

## Discussion

### Phylogenetic Analysis Indicates a Novel *Pseudomonas* sp.

Many plant-associated *Pseudomonas* strains show a diverse potential of promoting plant growth and acting as antagonists toward plant pathogens with a pivotal role in the plant microbiome. At the same time, a thorough genetic and functional characterization is important to discriminate beneficial strains from pathogenic members of the genus *Pseudomonas*. Our whole genome sequence analysis revealed that the most closely related type strain of SCA7 is *P. helmanticiensis*, which was isolated from forest soil, indicating an association with plants (Ramírez-Bahena et al., 2014). *P. helmanticiensis* is able to solubilize inorganic phosphate, which was tested but not observed for SCA7 (data not shown). Although the phylogenetic analysis of SCA7 revealed a dDDH value above the species delineation threshold of 70% (Goris et al., 2007), with the genome of *P. helmanticiensis*, SCA7 cannot unambiguously assigned to that species, because the ANI values with the closest related type strains were below the currently accepted species delineation threshold of 94% (Sangal et al., 2016). However, ANI values above 94% were identified with *P. koreensis* CFBP13504 and *P. koreensis* CI12, which are not type strains. According to a recent study disentangling the taxonomy of the *Pseudomonas stutzeri* species complex, ANI values were considered more important to assign species compared to dDDH values (Li et al., 2022). However, in their case, ANI values were clearly above the species delineation threshold of 94%, whereas dDDH values were below 70%. Nevertheless, our findings could indicate that SCA7 is a member of the species *P. helmanticiensis* or of a yet non-described *Pseudomonas* species, which could eventually include *P. koreensis* CFBP13504 identified in a metagenomics analysis from radish seeds (Torres-Cortés et al., 2019) and *P. koreensis* CI12 isolated from soybean rhizosphere (Lozano et al., 2017). Further phenotypic comparison to the different *Pseudomonas* type strains could support the correct assignment. The rhizosphere is also a reservoir of potential plant and human pathogens (Mendes et al., 2013). Therefore, it is of special importance to discriminate between beneficial and pathogenic bacteria for a safe use of PGPB in agricultural applications (Hartmann et al., 2019; Rodriguez et al., 2019). The phylogenetic classification of SCA7 revealed that SCA7 belongs to a group of plant-associated *Pseudomonas* and indicates that SCA7 is not closely related to plant or human pathogens. Several virulence factors (VFs), e.g., involved in flagella biosynthesis and motility, cell adherence, iron uptake, lipopolysaccharide biosynthesis, or regulatory proteins, have been detected in the SCA7 genome (Supplementary Table S6). VFs can be separated into common and pathogen-specific VFs, indicating the high similarity of host-microbe-interaction mechanisms of beneficial and pathogenic bacteria, where the pathogen-specific VFs are exclusively found in pathogens directly involved in virulence (Niu et al., 2013). Consistently, none of these pathogen-specific VFs were detected in SCA7. Instead, all identified VFs belong to the group of common VFs, which comprises microbial properties (i.e., gene products) frequently found in pathogenic as well as non-pathogenic bacteria, enabling microorganisms to interact and colonize the host. Additionally, we did not detect any disease symptoms on *A. thaliana* and *T. aestivum* plants inoculated with SCA7 in the conducted experiments. Taken together, the whole genome sequence analysis indicated that SCA7 is, rather, a non-pathogenic *Pseudomonas* strain and revealed the remarkable genomic potential of SCA7 in terms of beneficial plant-microbe interactions, as well as antagonistic activity against pathogens. The identified genes are discussed in the following paragraphs.

### SCA7 Enhances Plant Growth of *Arabidopsis thaliana* and Wheat (*Triticum aestivum*) and Reveals Plant Beneficial Traits *in vitro*

During a thorough characterization of the novel *Pseudomonas* strain SCA7, we showed that SCA7 displays several traits common to plant growth-promoting bacteria. SCA7 has a clear swarming motility *in vitro* and harbors genes involved in flagella biosynthesis and motility, suggesting that SCA7 is able to move toward and along the plant root for efficient colonization, as it has been previously demonstrated, e.g., for the PGPB *Bacillus subtilis* SWR01 (Gao et al., 2016; Blake et al., 2021). Furthermore, SCA7 produced biofilms *in vitro* in a static batch culture and was also detected using FISH to effectively colonize *A. thaliana* roots on the surface, mainly in zones of emerging root hairs. Root hairs are a common area for colonization by PGPB, because they deliver a high amount of root exudates, including important nutrients for bacterial growth (Prieto et al., 2011). Bacterial biofilm production is often mediated by quorum sensing, which depends, e.g., on N-acylated homoserine lactones (AHLs) (Fazli et al., 2014). However, biofilm production in SCA7 seems to be differently regulated, because genes involved in AHL production could not be clearly identified, and the AHL biosensor assay was negative. Instead, the SCA7 genome harbors genes encoding for the two-component regulatory system GacA/GacS, which are involved in small RNA signaling, presenting an additional system for concerted bacterial actions and also relevant to biofilm production in *Pseudomonas putida* and *Pseudomonas fluorescens* (Fazli et al., 2014). Interestingly, the SCA7 genome harbors genes encoding for transcriptional regulators of the LuxR family. As AHL production for SCA7 was negative, this regulator is most likely a so-called *luxR*-solo, which is able to sense AHL-signals of neighboring cells without contributing to signaling (Hartmann et al., 2021). Additionally, a gene encoding for the enzyme acyl-homoserine lactone acylase was identified, which is able to degrade AHLs (Rodríguez et al., 2020). Presence of these genes could indicate the ability of SCA7 to sense and interrupt AHL-based bacterial communication by quorum quenching (Dong et al., 2001). However, functionality of the quorum quenching ability needs to be further investigated. Taken together, targeted movement and localized biofilm formation at nutrient-rich areas of the rhizosphere clearly indicate the rhizosphere competence of SCA7.

SCA7 has the ability to modulate root architecture and to increase the fresh weight of the model organism *A. thaliana* in axenic conditions, as well as to increase the fresh weight of its original monocot host *T. aestivum* in a sand/clay system. These traits are known to be common for PGPB (Spaepen et al., 2007; Zamioudis et al., 2013), as demonstrated in the case of the plant growth-promoting strain *P. simiae* WCS417, which enhances plant growth and alters the root structure in *A. thaliana* (Zamioudis et al., 2013; Pieterse et al., 2020). Alteration of plant hormone homeostasis by plant beneficial bacteria has been reported to influence plant growth and development, including germination and flowering (Zamioudis et al., 2013; Panke-Buisse et al., 2017; Salazar-Cerezo et al., 2018; Finkel et al., 2020). Specifically, the production of the growth regulator auxin by bacteria has been shown to modulate root architecture, with an increased root surface for improved water and nutrient uptake (Spaepen and Vanderleyden, 2011). *Pseudomonas* strains, such as *P. simiae* WCS417 (Zamioudis et al., 2013), *P. fluorescens* Sasm05 (Chen et al., 2017) or *P. aeruginosa* TO3 (Khare and Arora, 2010), produce IAA, indicating that this trait is common in the genus *Pseudomonas*. In this study, we identified genes in the SCA7 genome involved in the production of an IAA precursor, tryptophan, and we showed that SCA7 can produce IAA, although less than the positive control *H. frisingense* GSF30. Thus, it is plausible that the observed increase in plant biomass, together with alterations in root structure observed in plants treated with SCA7, is partly related to IAA production by SCA7, strengthening, hence, the PGP potential for this isolate. Surprisingly, the auxin transport genes in *A. thaliana* did not show alterations, indicating that the effect on root architecture is related to the exogenous IAA produced by the bacteria, but without an effect on the endogenous IAA uptake pathway of the plant. However, gene expression was evaluated only after 24 h, which cannot exclude an effect on the endogenous IAA uptake pathway at a later time point. Additionally, bacterial root colonization is a sequential process with several phases from microcolonies to mature biofilm formation, which can take longer than 24 h, and this can influence amounts of bacterial-produced plant hormones (Knights et al., 2021). If a certain concentration of bacterial IAA is required to observe a molecular reaction in the plant, the amount of IAA might have been below the required limit, which leads to the observed lack of alterations in expression of auxin transport genes. Nevertheless, also, other hormones, such as cytokinins and gibberellins, are involved in root development and can be produced by bacteria (Bottini et al., 2004; Kudoyarova et al., 2019).

PGPB can stimulate plant productivity by improving plant nutrient acquisition (Glick, 2012). Here, we investigated the early plant root responses to cultivation with SCA7 by profiling some marker genes for the uptake or response to several nutrients. Nitrate and phosphate are highly demanded to form plant biomass (Pandey, 2015; Ueda et al., 2020). In this study, we showed that, after 24 h incubation with SCA7, the expression of gene *NRT2.1* was upregulated in *A. thaliana* roots, whereas *PHT1.1* was not. This indicates that only transport of nitrogen was activated. The upregulation of *IRT1* and *MYB72*, relevant to iron uptake, was even stronger than the upregulation of the nitrogen transport genes in *A. thaliana* roots 24 h after inoculation with SCA7. Interestingly, this response resembles *P. simiae* WCS417-induced iron deficiency response, which includes the activation of IRT1 and MYB72 in *A. thaliana* roots, albeit at a later time point (48 h) (Verbon et al., 2019). This bacteria-induced iron deficiency response has been shown to elevate iron uptake in the shoots and improve plant growth under iron-sufficient conditions (Verbon et al., 2019). However, the reaction was not caused by an actual iron shortage, e.g., *via* production of iron-chelating siderophores by the bacteria, because mutant bacterial lines impaired in siderophore production and the exposition of plants to mVOCs produced by ISR-inducing microbes showed the same effect of improved iron status in the plant (Zamioudis et al., 2015; Martínez-Medina et al., 2017). The actual mechanism of the iron deficiency response is, to date, unknown (Verbon et al., 2019). Aside from this special case, siderophore production in bacteria can have a beneficial effect on the plant's iron status but is dependent on the compatibility of the specific siderophore with the receptors of the plant and can vary between cultivars, as shown for the siderophore pyoverdine and pea plants (Lurthy et al., 2020). The siderophore pyoverdine is common in plant-associated *Pseudomonas* (Berendsen et al., 2015) and also produced by *P. simiae* WCS417, which enhances plant growth in *A. thaliana* and is effective against fungal pathogens in competition for iron (Pieterse et al., 2020). The presence of genes encoding for pyoverdine and other siderophores in the SCA7 genome and the actual SCA7 siderophore production *in vitro* indicate the potential of SCA7 to modify its surrounding by regulation of iron availability, benefitting itself and/or its host. In line with this, improving iron nutrition in plants by PGPB has been linked to their ability to trigger ISR, for example, but not exclusively, by the structure of specific siderophores (Berendsen et al., 2015; Romera et al., 2019). Here, we found that expression of the pathogenesis-related gene *PR1* is slightly downregulated or unchanged in root tissue inoculated with SCA7. A similar trend was observed after expression analysis of MYC2 TF, also relevant for pathogen responses. Thus, these results provide an insight into the molecular mode of action of SCA7, which, indeed, resembles the well-studied PGPB *P. simiae* WCS417 by triggering an iron deficiency response, causing the activation of iron transporters mediated by activation of *MYB72* (Verbon et al., 2019). Moreover, we also observed that SCA7 is not associated with upregulation of defense responses under unchallenged growth conditions but only after subsequent exposure to the foliar pathogen *Pst DC3000*, as described in the next sections. This shows that the plant does not recognize SCA7 as pathogenic and, instead, indicates a priming response of the plant (Martinez-Medina et al., 2016).

To establish such a mutualistic interaction with the plant, a PGPB is likely equipped with active suppression mechanisms of local root immune responses. One way to facilitate this would be *via* type-3-secretion systems (T3SSs) and T3SS-effector proteins (Stringlis et al., 2019; Saad et al., 2020). The plant beneficial *P. fluorescens* 2P24 (Liu et al., 2016) and *P. simiae* WCS417 (Stringlis et al., 2019), together with many plant-pathogenic gram-negative bacteria, possess, indeed, a T3SS. In our study, we identified genes encoding for parts of type-6-secretion systems frequently found in non-pathogenic bacteria (Niu et al., 2013), the channel protein secretin, and the effector protein HopPmaJ in the SCA7 genome, but not genes encoding for the needle complex required for the translocation of effector proteins. HopPmaJ is a T3SS-effector protein found in several pathogenic *Vibrio* bacterial species and relevant to bacterial virulence (Zhao et al., 2017; Zhuang et al., 2020) and secretin, although is known to be involved in the type-2-secretion system, suggesting that SCA7 might not possess a regular T3SS. However, the possibility of other effector delivery systems in SCA7, such as secretin-forming membrane channels (Type-2-secretion system) or type-6-secretion systems, together with the presence of other effector proteins, should not be excluded.

### SCA7 Shows Antagonistic Interactions Against Plant-Pathogenic Microorganisms

In the rhizosphere complex, multitrophic interactions take place among microorganisms and host plants, as well as among individual microorganisms of the same microbial community. Mechanisms involved in antagonistic effects of beneficial bacteria against pathogens could involve secondary metabolites or compounds, which are advantageous for beneficial bacteria in the competition for the same resources, such as siderophores for iron scavenging (Gu et al., 2020). Many species of *Pseudomonas* are, indeed, potential biocontrol agents (Haas and Keel, 2003) and are known for their production of secondary metabolites, which are involved in antagonistic mechanisms against plant-pathogenic fungi and oomycetes (Johnsson et al., 1998; De Vrieze et al., 2018; Tagele et al., 2019). Secondary metabolites, often encoded by biosynthetic gene clusters (BGCs), can influence microbe-microbe and microbe-plant interactions. Here, we showed that SCA7 has the ability to inhibit the growth of three different plant-pathogenic fungi, and 11 biosynthetic gene clusters (BGCs) were identified in SCA7 in this study, including the BGC arylpolyene Vf, which is involved in, e.g., antifungal activity (Dutta et al., 2020). Phenazine is another common antifungal compound among *Pseudomonas* species (Yu et al., 2018; Tagele et al., 2019), together with the cyclic lipopeptide lokisin (Omoboye et al., 2019). In the genome of SCA7, we identified genes involved in the biosynthesis of phenazine and the BGC for lokisin with high similarity to the described gene clusters. Thus, it is plausible that the ability of SCA7 to halt pathogenic fungal growth is due to the production of antifungal secondary metabolites, also identified previously in other *Pseudomonas* species. However, it was beyond the scope of this study to prove the presence of these compounds in the used media. Apart from antifungal properties, members of the genus *Pseudomonas* were reported to also suppress bacterial pathogens (Hu et al., 2016), and genes involved in the biosynthesis of antibacterial secondary metabolites, such as colicin V, lankacidin C, and other bacteriocins, were identified in the genome of SCA7 in this study. Colicin V is a peptide antibiotic secreted by some Enterobacteriacea, used to kill closely related bacterial cells by disrupting their membrane potential (Gérard et al., 2005; Cascales et al., 2007). Structurally, similar antibiotics in the genus *Pseudomonas* are called pyocins (Cascales et al., 2007; Ghequire and De Mot, 2014). The lankacidin C BGC identified in SCA7 had a rather low similarity with lankacidin C from the soil-dwelling bacteria *Streptomyces rochei*, which is active against gram-positive bacteria (Harada et al., 1969). Lankacidin C was also identified in the strain *Pseudomonas kilonensis* F113 (Rieusset et al., 2020). Thus, the antagonistic effect of SCA7 against the tested pathogenic bacterial strains might be, at least, partly caused by the presence of secreted secondary metabolites. It is clear that effective antibacterial secondary metabolites are not selectively effective against plant pathogens and, therefore, can also inhibit non-pathogenic bacteria, which was also observed in a pre-experiment with SCA7 (data not shown). If this inhibiting activity of SCA7 causes transient or long-term microbiome shifts and if these shifts actually influence crop production need to be further investigated. However, SCA7 was isolated from a natural community, showed a positive effect on plant growth, and, when confronting SCA7 with the plant beneficial bacterial strain *Bacillus velezensis* FZB42, we did not observe growth reduction of any of the two strains, although also FZB42 produces very effective antibacterial compounds (Chowdhury et al., 2015). This observation proved SCA7's compatibility with the known PGPB, *Bacillus velezensis* FZB42, making it suitable for mixed inoculations. Taken together, SCA7 has the genomic and functional potential to mount diverse antagonistic microbial interactions by producing specific secondary metabolites with antifungal or antibacterial properties.

Antimicrobial activity of SCA7 was also observed in the two-compartment (a split plate) system despite the physical barrier between the tested microorganisms. This effect might be related to volatile compounds, which are known to have several ecological functions, such as plant growth promotion and biocontrol or cross-talk between plants and microbes (Netzker et al., 2020; Berg et al., 2021). Microbial VOCs can induce plant growth, e.g., by modulation of essential nutrients, hormonal balance, metabolism, and sugar concentrations (Fincheira and Quiroz, 2018). The compounds 1-undecene and 2,3-butanediol were reported to enhance plant growth and to be involved in biocontrol mechanisms (Lo Cantore et al., 2015; Fincheira and Quiroz, 2018; Tagele et al., 2019; Netzker et al., 2020). Genes encoding for both compounds were identified in the SCA7 genome, and our results of GC-MS analysis revealed the production of the antifungal compounds octanal and 1-undecene (Netzker et al., 2020) by SCA7 under unchallenged conditions. This baseline production of antimicrobial volatiles might be even extended in confrontation with pathogens (Netzker et al., 2020). Therefore, the antagonistic activity of SCA7 against the tested microorganisms could be due to the ability of SCA7 to produce mVOCs with antagonistic effects against plant pathogens, which could be interesting for potential biocontrol applications over distances, e.g., for inhibition of the aboveground pathogens, such as *Pst* DC3000, by root-inoculated antagonists, such as SCA7.

Inoculation of *A. thaliana* roots growing in soil with SCA7 significantly reduced the reproduction of the pathogen *Pst* DC3000 in infected leaves and confirmed that SCA7 exerts an indirect effect on plant defense response. Induced systemic resistance (ISR) in healthy tissues by long-distance signaling of distally inoculated beneficial microbes is a well-studied phenomenon (Pieterse et al., 2014). Based on these observations, we assumed that the antibacterial effect of SCA7 against *Pst* DC3000 in *A. thaliana* plants involves the production of mVOCs with antibacterial properties (discussed above) and/or the activation of ISR. We showed that ISR is activated by SCA7 inoculation in roots *via* upregulation of the TF *MYB72*. During pathogen attack by *Pst* DC3000, defense responses related to SA and JA/ET signaling pathways are strongly upregulated in SCA7-inoculated plants than in uninoculated plants. Thus, SCA7 shows similar activity against the pathogen *Pst* DC3000 as other *Pseudomonas* spp., such as WCS417, WCS358, and SS101 (Van Oosten et al., 2008; van de Mortel et al., 2012; Pangesti et al., 2017). Interestingly, these *Pseudomonas* spp. can also induce ISR against other pathogens, such as the fungus *Fusarium oxysporum* or even to the generalist herbivore *Spodoptera exigua* (Van Oosten et al., 2008). Thus, we could speculate that our novel found *Pseudomonas* sp. SCA7 could also display a wide range of biocontrol effects against other pathogens, besides the here tested *Pst* DC3000. Despite similar protection effects like hampering pathogen growth, the mechanisms of defense response activation by beneficial bacteria do not necessarily have to be the same (van de Mortel et al., 2012; Pieterse et al., 2020). For instance, *P. fluorescens* SS101-induced resistance to *Pst* DC3000 depends on SA signaling and not on JA or ET signaling. In contrast, we observed here that signaling based on all these hormones is involved in response to SCA7 inoculation.

## Conclusion

In this study, we identified SCA7 as a potential member of a novel *Pseudomonas* species. Through a thorough and comprehensive characterization, we showed that SCA7 displays promising PGP activities and a potential broad host range, indicated by the similar effects on the dicot *A. thaliana* and the monocot *T. aestivum*. Moreover, we showed that SCA7, as other PGPB, shows antimicrobial activities *in vitro* and has the ability of triggering ISR against a foliar biotrophic pathogen, thus protecting its host against biotic stresses. These plant beneficial traits make SCA7 a suitable candidate for further large-scale studies on plants in more complex settings with natural soils in growth chambers, as well as in the field. These studies would reveal the optimal SCA7 application conditions for different crop plants and settings to pave the way for the development of an effective biocontrol agent.

## Data Availability Statement

The datasets presented in this study can be found in online repositories. The names of the repository/repositories and accession number(s) can be found below: https://www.ncbi.nlm.nih.gov/, NZ_CP073104.

## Author Contributions

TK-N and PR contributed to conception and design of the study and wrote the first draft of the manuscript. TK-N performed the genomic analyses and contributed parts of the experimental data. IG performed *in vitro* and plant experiments. MR, BW, and J-PS performed the GC-MS including data analysis. SK sequenced the genome. PR, TK-N, IG, SC, and PS analyzed the data. SC, MR, MS, and PF-B wrote sections of the manuscript. All authors contributed to manuscript revision, read, and approved the submitted version.

## Conflict of Interest

TK-N, PR, IS, SP, PS, MR, BW, J-PS, SK, MS, MR, and PF-B were employed by Helmholtz Center Munich, German Research Center for Environmental Health.

## Publisher's Note

All claims expressed in this article are solely those of the authors and do not necessarily represent those of their affiliated organizations, or those of the publisher, the editors and the reviewers. Any product that may be evaluated in this article, or claim that may be made by its manufacturer, is not guaranteed or endorsed by the publisher.
