# Supplementary material for: Novel Pseudomonas sp. SCA7 Promotes Plant Growth in Two Plant Families and Induces Systemic Resistance in Arabidopsis thaliana
Source: Front Microbiol. 2022 Jun 27;13:923515. doi: 10.3389/fmicb.2022.923515 (PMC9297469; doi:10.3389/fmicb.2022.923515)
Supplement: Supplementary file 1 [file Data_Sheet_1.docx]

Supplementary Material

# Supplementary Table

**Table S1**: qRT-PCR primer pairs used in this study.

| **Gene Name** | **GeneID** | **Primer** | **Sequence 5'–3'** | **Ref.** |
| --- | --- | --- | --- | --- |
|  |  | **Primer pairs for qRT-PCR with shoot material** | |  |
| *EF1* | At5g60390 | EF-1α_F | TGAGCACGCTCTTCTTGCTTTCA | Czechowski et al., 2005 |
|  |  | EF-1α_R | GGTGGTGGCATCCATCTTGTTACA |  |
| *JAZ10* | At5g13229 | JAZ10.1_F | GAAGCGCAAGGAGAGATTAG | Schmiesing et al., 2016 |
|  |  | JAZ10.1_R | CAACGACGAAGAAGGCTTCA |  |
| *PDF1.2* | At5g44420 | Pdf1.2_F | CTTGTTCTCTTTGCTGCTTTCGAC | Poupin et al., 2013 |
|  |  | Pdf1.2_R | ATGCATTACTGTTTCCGCAAACC |  |
| *PR1* | At2g14610 | PR1_F | CGGAGCTACGCAGAACAACT | Piisilä et al., 2015 |
|  |  | PR1_R | AAGGCCCACCAGAGTGTATG | Kim et al., 2016 |
| *PR2* | At3G57260 | PR2_F | ATCTCCCTTGCTCGTGAATCTCT | Liu et al., 2013 |
|  |  | PR2_R | TCGAGATTTGCGTCGAATAGG |  |
| *PR5* | At1G75040 | PR5_F | GAGGATCGGGAGATTGCAAA | Liu et al., 2013 |
|  |  | PR5_R | CTCCACGGCAGCAATATTGA |  |
| *SID2* | At1G74710 | SID2_F | CCCTTAACAAGGTTGTTCTTGC | Gao et al., 2013 |
|  |  | SID2_R | CCTTCACGCTGTAACTGTGC |  |
| *VSP2* | At5g24770 | VSP2_F | GTTAGGGACCGGAGCATCAA | Schmiesing et al., 2016 |
|  |  | VSP2_R | TCAATCCCGAGCTCTATGATGTT |  |
| *WRKY70* | At3g56400 | WRKY70_F | ACTTGAGGACGCATTTTCTTGGAGG | Zhou et al., 2011 |
|  |  | WRKY70_R | TGCTTTGTTGCCTTGCACCCTT |  |
| *MYC2* | AT1G32640 | MYC2_F | TCATACGACGGTTGCCAGAA | Carvalhais et al., 2013 |
|  |  | MYC2_R | AGCAACGTTTACAAGCTTTGATTG |  |
| *IAA1* | AT4G14560 | IAA1_F | TGGAAGTCACCAATGGGCTTAACCT | Poupin et al., 2013 |
|  |  | IAA1_R | TGCGCTTGTTGTTGCTTCTGACG |  |
|  |  | **Primer pairs for qRT-PCR with root material** | |  |
| *Actin7* | At2G14610 | ACTIN7_F | AGTGGTCGTACAACCGGTATTGT | Zamioudis et al. 2014 |
|  |  | ACTIN7_R | GATGGCATGAGGAAGAGAGAAAC |  |
| *AUX1* | At2G38120 | AUX1_F | ACTTCTCGACCACTCCAACGC | Li et al., 2011 |
|  |  | AUX1_R | ATCCCAATCACTTTCTCCCAC |  |
| *LAX3* | At1G77690 | LAX3_F | TCACCATTGCTTCACTCCTTC | El-Showk et al., 2015 |
|  |  | LAX3_R | AAGCACCATTGTGGTTGGAC |  |
| *IAA1* | AT4G14560 | IAA1_F | TGGAAGTCACCAATGGGTTTAACCT | Poupin 2016 |
|  |  | IAA1_R | TGCCGTTGTTGTTGCTTATGACG |  |
| *NRT2.1* | At1G08090 | NRT2.1_F | AAAGACAAATTCGGAAAGATTCTG | Mantelin et al. 2006 |
|  |  | NRT2.1_R | AAGTACTCGGCGATAACATTATCA |  |
| *IRT1* | AT4G19690 | IRT1_F | ACCCGTGCGTCAACAAAGCTAAAG | Zamioudis et al. 2014 |
|  |  | IRT1_R | TCCCGGAGGCGAAACACTTAATGA |  |
| *PHT1.1* | At5G43350 | PHT1;1_F | AGAGCCCAAAGGCAAGTCC | Ueda et al., 2020 |
|  |  | PHT1;1_R | AACATCATAACTTAAGGTCAACGAGC |  |
| *MYB72* | At1g56160 | MYB72_F | ACGAGATCAAAAACGTGTGGAAC | Zamioudis et al. 2014 |
|  |  | MYB72_R | TCATGATCTGCTTTTGTGCTTTG |  |
| *BGLU* | AT5G36890 | BGLU42_F | ATGGCCTGGGAACTGAAGTC | Zamioudis et al. 2014 |
|  |  | BGLU42_R | ATTTGTCCAACCTCCGATTG |  |
| *MYC2* | AT1G32640 | MYC2_F | TCATACGACGGTTGCCAGAA | Carvalhais et al., 2013 |
|  |  | MYC2_R | AGCAACGTTTACAAGCTTTGATTG |  |
| *PR1* | At2g14610 | PR1_F | CGGAGCTACGCAGAACAACT | Piisilä et al., 2015 |
|  |  | PR1_R | AAGGCCCACCAGAGTGTATG | Kim et al., 2016 |

**References**

Kim, M.K., Yeo, B.E., Park, H., Huh, Y.D., Kwon, C., Yun, H.S. (2016) Dual effect of the cubic Ag3PO4 crystal on *Pseudomonas syringae* growth and plant immunity. *Plant Pathol J*. 32(2):168-70. doi: 10.5423/PPJ.NT.09.2015.0191.

Zamioudis, C., Hanson, J., Pieterse, C.M. (2014). β-Glucosidase BGLU42 is a MYB72-dependent key regulator of rhizobacteria-induced systemic resistance and modulates iron deficiency responses in Arabidopsis roots. *New Phytol*. 204(2):368-79. doi: 10.1111/nph.12980.

Zhou, X., Jiang, Y., & Yu, D. (2011). WRKY22 transcription factor mediates dark-induced leaf senescence in Arabidopsis. *Mol. Cells*, 31(4), 303–313. https://doi.org/10.1007/s10059-011-0047-1

Liu, W.X., Zhang, F.C., Zhang, W.Z., Song, L.F., Wu, W.H., Chen, Y.F. (2013). Arabidopsis Di19 functions as a transcription factor and modulates PR1, PR2, and PR5 expression in response to drought stress. *Mol Plant*.6(5):1487-502. doi: 10.1093/mp/sst031.

Poupin, M.J., Timmermann, T., Vega, A., Zuñiga, A., González, B. (2013) Effects of the plant growth-promoting bacterium *Burkholderia phytofirmans* PsJN throughout the life cycle of *Arabidopsis thaliana*. *PLoS ONE* 8(7): e69435. <https://doi.org/10.1371/journal.pone.0069435>

Schmiesing, A., Emonet, A., Gouhier-Darimont, C., & Reymond, P. (2016). Arabidopsis MYC Transcription Factors Are the Target of Hormonal Salicylic Acid/Jasmonic Acid Cross Talk in Response to Pieris brassicae Egg Extract. *Plant physiology*, 170(4), 2432–2443. https://doi.org/10.1104/pp.16.00031

Piisilä, M., Keceli, M.A., Brader, G. et al. (2015). The F-box protein MAX2 contributes to resistance to bacterial phytopathogens in *Arabidopsis thaliana*. *BMC Plant Biol* 15, 53. https://doi.org/10.1186/s12870-015-0434-4

Czechowski, T., Stitt, M., Altmann, T., Udvardi, M.K., Scheible, W.R. (2005). Genome-wide identification and testing of superior reference genes for transcript normalization in Arabidopsis. *Plant Physiol*. 139(1):5-17. doi: 10.1104/pp.105.063743.

El-Showk, S., Help-Rinta-Rahko, H., Blomster, T., Siligato, R., Marée, A.F.M., et al. (2015) Parsimonious Model of Vascular Patterning Links Transverse Hormone Fluxes to Lateral Root Initiation: Auxin Leads the Way, while Cytokinin Levels Out. *PLOS Computational Biology* 11(10): e1004450. https://doi.org/10.1371/journal.pcbi.1004450

Poupin, M.J., Greve, M., Carmona, V. and Pinedo, I. (2016) A Complex Molecular Interplay of Auxin and Ethylene Signaling Pathways Is Involved in Arabidopsis Growth Promotion by Burkholderia phytofirmans PsJN. *Front. Plant Sci.* 7:492. doi: 10.3389/fpls.2016.00492

Carvalhais Lilia, Muzzi Frederico, Tan Chin-Hong, Choo Jin Hsien, Schenk Peer (2013), Plant growth in Arabidopsis is assisted by compost soil-derived microbial communities, Frontiers in *Plant Science*, 4:235, doi:10.3389/fpls.2013.00235

Gao, X., Chen, X., Lin, W., Chen, S., Lu, D., et al. (2013) Bifurcation of Arabidopsis NLR immune signaling via Ca2+-dependent protein kinases. *PLOS Pathogens* 9(1): e1003127. https://doi.org/10.1371/journal.ppat.1003127

Mantelin, S., Desbrosses, G., Larcher, M., Tranbarger, T. J., Cleyet-Marel, J.-C., & Touraine, B. (2006). Nitrate-dependent control of root architecture and N nutrition are altered by a plant growth-promoting *Phyllobacterium* sp. *Planta*, 223(3), 591–603. http://www.jstor.org/stable/23389233

**Table S2**: Genome properties of SCA7

| Genome properties | SCA7 |
| --- | --- |
| Chromosome size (Mbp) | 6.78 |
| GC content (%) | 59.1 |
| Coding Sequences (PGAP) | 5981 |
| rRNAs 16S | 6 |
| NCBI Accession Number | NZ_CP073104 |

**Table S3**: List of annotated genes in *Pseudomonas* sp. SCA7 with the Software antiSMASH.

|  | ***Type*** | ***From (bp)*** | ***To (bp)*** | ***Most similar known cluster*** | ***Class of secondary metabolites*** | ***Similarity*** | ***comment/category*** |
| --- | --- | --- | --- | --- | --- | --- | --- |
| *1* | *NRPS* | 60,885 | 137,783 | lokisin | NRP | 85% | Antifungal compounds |
| *2* | *siderophore* | 1,322,522 | 1,334,372 |  |  |  | siderophore |
| *3* | *RiPP-like* | 1,537,456 | 1,547,441 |  |  |  | multiple functions |
| *4* | *NRPS* | 1,816,323 | 1,888,411 | pyoverdin | NRP | 18% | Siderophore/Antifungal compounds |
| *5* | *RiPP-like* | 2,669,750 | 2,680,637 |  |  |  | multiple functions |
| *6* | *arylpolyene* | 3,653,220 | 3,696,824 | APE Vf | Other | 40% | biocontrol in general, most common group of BGC in proteobacteria, biofilm formation |
| *7* | *NRPS-like* | 4,008,341 | 4,038,506 | fragin | NRP | 37% | Antifungal compounds |
| *8* | *redox-cofactor* | 4,743,426 | 4,765,582 | lankacidin C | NRP + Polyketide | 13% | antibiotic |
| *9* | *NAGGN* | 5,960,677 | 5,975,567 |  |  |  | osmotic stress |
| *10* | *NRPS* | 6,115,849 | 6,168,847 | pyoverdin | NRP | 17% | Siderophore/Antifungal compounds |
| *11* | *betalactone* | 6,507,383 | 6,530,646 | fengycin | NRP | 13% | Antifungal compounds |

**Table S4**: List of annotated genes in Pseudomonas sp. SCA7 after functional annotation with the software RAST (Version 2.0; Aziz et al., 2008).

| **category/function** | **Annotated Genes and Cluster** | **SCA7 gene ID (PGAP)** |
| --- | --- | --- |
| **Auxin biosynthesis** | Tryptophan synthase alpha chain (EC 4.2.1.20) | KBP52_17925 |
|  | Tryptophan synthase beta chain (EC 4.2.1.20) | KBP52_17930 |
|  | Anthranilate phosphoribosyltransferase (EC 2.4.2.18) | KBP52_21430 |
|  | Phosphoribosylanthranilate isomerase (EC 5.3.1.24) | KBP52_07890 |
| **mVOCs production** | |  |
| Butanediol metabolism/synthesis | Acetolactate synthase large subunit (EC 2.2.1.6) | KBP52_23150 |
|  | Acetolactate synthase small subunit (EC 2.2.1.6) | KBP52_23155 |
| 1-Undecene | Putative 1-Undecene synthesis gene undA: Pyrroloquinoline quinone (Coenzyme PQQ) biosynthesis protein C | KBP52_26850 |
| **Siderophore production** | |  |
| Siderophore Pyoverdine biosynthetic gene cluster | Pyoverdine chromophore precursor synthetase PvdL | KBP52_27520 |
|  | Pyoverdine efflux carrier and ATP binding protein | KBP52_08140 |
|  | FIG049111: Hypothetical protein in pyoverdin gene cluster | KBP52_27595 |
|  | Outer membrane pyoverdine eflux protein | KBP52_08145 |
|  | FIG006220: Hypothetical MbtH-like protein | KBP52_27570 |
|  | Outer membrane porin, coexpressed with pyoverdine biosynthesis regulon | KBP52_23820 |
|  | Outer membrane ferripyoverdine receptor | KBP52_01340 |
|  | ABC transporter in pyoverdin gene cluster, periplasmic component | KBP52_27575 |
|  | Sigma-70 factor FpvI (ECF subfamily), controling pyoverdin biosynthesis | KBP52_28410 |
|  | Putative dipeptidase, pyoverdin biosynthesis PvdM | KBP52_08155 |
|  | PvdO, pyoverdine responsive serine/threonine kinase (predicted by OlgaV) | KBP52_08165 |
|  | Non-ribosomal peptide synthetase modules, pyoverdine | KBP52_08185, KBP52_08195 |
|  | Pyoverdine sidechain non-ribosomal peptide synthetase PvdD | KBP52_08190 |
|  | FIG014801: Cation ABC transporter, periplasmic cation-binding protein | KBP52_27590 |
|  | pyoverdine-specific efflux macA-like protein | KBP52_08135 |
|  | Sigma factor PvdS, controling pyoverdin biosynthesis | KBP52_27515 |
|  | ABC transporter in pyoverdin gene cluster, permease component | KBP52_27580 |
|  | Hypothetical protein PvdY | KBP52_27510 |
|  | Pyoverdin biosynthesis protein PvdH, L-2,4-diaminobutyrate:2-oxoglutarate aminotransferase (EC 2.6.1.76) | KBP52_27565 |
|  | FIG139991: Putative thiamine pyrophosphate-requiring enzyme | KBP52_27605 |
|  | L-ornithine 5-monooxygenase (EC 1.13.12.-), PvdA of pyoverdin biosynthesis | KBP52_08125 |
|  | PvdE, pyoverdine ABC export system, fused ATPase and permease components | KBP52_08170 |
|  | ABC transporter in pyoverdin gene cluster, ATP-binding component | KBP52_27580 |
|  | FIG137877: Hypothetical protein in pyoverdin gene cluster | KBP52_27600 |
|  | Pyoverdine biosynthesis related protein PvdP | KBP52_08150 |
|  | Pyoverdin biosynthesis protein PvdN, putative aminotransferase, class V | KBP52_08160 |
| siderophore Achromobactin biosynthetic gene cluster | TonB-dependent ferric achromobactin receptor protein | KBP52_03145 |
| Iron siderophore sensor and receptor system | FIG006045: Sigma factor, ECF subfamily | KBP52_08130, KBP52_13560, KBP52_13580, KBP52_18480, KBP52_25340, KBP52_28410 |
|  | Iron siderophore receptor protein | KBP52_13590, KBP52_28420 |
|  | Iron siderophore sensor protein | KBP52_02535, KBP52_05035, KBP52_13555, KBP52_13585, KBP52_25335, KBP52_28415 |
| **Quorum sensing, quorum quenching and signaling** | Acyl-homoserine lactone acylase PvdQ (EC 3.5.1.-), quorum-quenching | KBP52_02530 |
|  | Transcriptional regulator, LuxR family | KBP52_23620, KBP52_24470 |
|  | BarA-associated response regulator UvrY (= GacA = SirA) | KBP52_04730 |
|  | BarA sensory histidine kinase (= VarS = GacS) | KBP52_26130 |
| **Antibiotic compounds** | |  |
| Colicin V and Bacteriocin Production cluster | Amidophosphoribosyltransferase (EC 2.4.2.14) | KBP52_07865 |
|  | Colicin V production protein | KBP52_07870 |
|  | Dihydrofolate synthase (EC 6.3.2.12) | KBP52_07880 |
|  | Acetyl-coenzyme A carboxyl transferase beta chain (EC 6.4.1.2) | KBP52_07885 |
|  | DedA protein | KBP52_22090, KBP52_22200 |
|  | DedD protein | KBP52_07875 |
|  | tRNA pseudouridine synthase A (EC 4.2.1.70) | KBP52_07895, KBP52_12230 |
| **Antifungal compounds** | |  |
| Putative Phenanzine biosynthetic cluster | PhnB protein | KBP52_02315 |
|  | Alkylphosphonate utilization operon protein PhnA | KBP52_22750 |
|  | PhnB protein; putative DNA binding 3-demethylubiquinone-9 3-methyltransferase domain protein | KBP52_27640 |
|  | Uncharacterized isomerase yddE, PhzC-PhzF family | KBP52_24585 |
| **Secretion System and effector proteins** |  |  |
| Secretion System | Secretin = Type II and III secretion system family protein FIG00955375 | KBP52_29415 |
| Effector protein | Type III effector HopPmaJ | KBP52_13785 |
| **Antibiotic resistance** | Vancomycin B-type resistance protein VanW | KBP52_07035 |
|  | Fosfomycin resistance protein FosA | KBP52_11560 |
|  | RND multidrug efflux transporter; Acriflavin resistance protein | KBP52_24765 |
|  | Acriflavin resistance plasma membrane protein | KBP52_02335 |
|  | Polymyxin resistance protein ArnT, undecaprenyl phosphate-alpha-L-Ara4N transferase; Melittin resistance protein PqaB | KBP52_14715, KBP52_03045 |
|  | Polymyxin resistance protein PmrM | KBP52_03035 |
|  | Polymyxin resistance protein PmrL, sucrose-6 phosphate hydrolase | KBP52_03040 |
|  | Polymyxin resistance protein PmrJ, predicted deacetylase | KBP52_03050 |
|  | UDP-4-amino-4-deoxy-L-arabinose formyltransferase (EC 2.1.2.13) / UDP-glucuronic acid oxidase (UDP-4-keto-hexauronic acid decarboxylating) (EC 1.1.1.305) | KBP52_03055 |
|  | Polymyxin resistance protein ArnC, glycosyl transferase (EC 2.4.-.-) | KBP52_03060 |
|  | UDP-4-amino-4-deoxy-L-arabinose--oxoglutarate aminotransferase (EC 2.6.1.-) | KBP52_03065 |
|  | Polymyxin resistance protein ArnT, undecaprenyl phosphate-alpha-L-Ara4N transferase; Melittin resistance protein PqaB | KBP52_03090 |
| **Stress resistance** | Osmotic stress (36)  (Choline and betaine uptake, Glucans and sacrosine biosynthesis) | multiple genes |
|  | Oxidative stress (95) | multiple genes |
|  | Cold shock (6) | multiple genes |
|  | Heat shock (17) | multiple genes |
|  | Detoxification (28) | multiple genes |
|  | Stress Response - no subcategory (36) | multiple genes |
|  | Periplasmic Stress (6) | multiple genes |
| **Flagella biosynthesis and motility** | Flagellar motor rotation protein MotB | KBP52_10250, KBP52_15915 |
|  | Flagellar motor rotation protein MotA | KBP52_10255, KBP52_15920 |
|  | Signal transduction histidine kinase CheA (EC 2.7.3.-) | KBP52_01090, KBP52_10265 |
|  | Chemotaxis regulator - transmits chemoreceptor signals to flagelllar motor components CheY | KBP52_04245, KBP52_10275 |
|  | Chemotaxis response regulator protein-glutamate methylesterase CheB (EC 3.1.1.61) | KBP52_10260 |
|  | Chemotaxis response - phosphatase CheZ | KBP52_10270 |
|  | Flagellar synthesis regulator FleN | KBP52_10285 |
|  | Flagellar biosynthesis protein FlhF | KBP52_10290 |
|  | Flagellar biosynthesis protein FlhA | KBP52_10295 |
|  | Flagellar biosynthesis protein FlhB | KBP52_10335 |
|  | Flagellar biosynthesis protein FliR | KBP52_10340 |
|  | Flagellar biosynthesis protein FliQ | KBP52_10345 |
|  | Flagellar biosynthesis protein FliP | KBP52_10350 |
|  | Flagellar biosynthesis protein FliO | KBP52_10355 |
|  | Flagellar motor switch protein FliN | KBP52_10360 |
|  | Flagellar motor switch protein FliM | KBP52_10365 |
|  | Flagellar biosynthesis protein FliL | KBP52_10370, KBP52_19810 |
|  | Flagellar hook-length control protein FliK | KBP52_10375 |
|  | Flagellar protein FliJ | KBP52_10400 |
|  | Flagellum-specific ATP synthase FliI | KBP52_10405 |
|  | Flagellar assembly protein FliH | KBP52_10410 |
|  | Flagellar motor switch protein FliG | KBP52_10415 |
|  | Flagellar M-ring protein FliF | KBP52_10420 |
|  | Flagellar hook-basal body complex protein FliE | KBP52_10425 |
|  | Flagellar regulatory protein FleQ | KBP52_10430, KBP52_10440 |
|  | Flagellar sensor histidine kinase FleS | KBP52_10435 |
|  | Flagellar biosynthesis protein FliS | KBP52_10450 |
|  | Flagellar hook-associated protein FliD | KBP52_10455 |
|  | Flagellin protein FlaG | KBP52_10460 |
|  | Flagellin protein FlaA | KBP52_10465 |
|  | Flagellar hook-associated protein FlgL | KBP52_10560 |
|  | Flagellar hook-associated protein FlgK | KBP52_10565 |
|  | Flagellar protein FlgJ [peptidoglycan hydrolase] (EC 3.2.1.-) | KBP52_10570 |
|  | Flagellar P-ring protein FlgI | KBP52_10575 |
|  | Flagellar L-ring protein FlgH | KBP52_10580 |
|  | Flagellar basal-body rod protein FlgG | KBP52_10585 |
|  | Flagellar basal-body rod protein FlgF | KBP52_10590 |
|  | Flagellar biosynthesis protein FlgN | KBP52_25965 |
|  | Negative regulator of flagellin synthesis FlgM | KBP52_25970 |
|  | Flagellar basal-body P-ring formation protein FlgA | KBP52_25975 |
|  | Chemotaxis protein CheV (EC 2.7.3.-) | KBP52_25980 |
|  | Chemotaxis protein methyltransferase CheR (EC 2.1.1.80) | KBP52_25985 |
|  | Flagellar basal-body rod protein FlgB | KBP52_25990 |
|  | Flagellar basal-body rod protein FlgC | KBP52_25995 |
|  | Flagellar basal-body rod modification protein FlgD | KBP52_26000 |
|  | Flagellar hook protein FlgE | KBP52_26005 |

**Table S5**: Gene homologs identified in the SCA7 genome using CARD based on the protein homolog model and strict resistance gene identifier (= a strict match is not identical but the bitscore of the matched sequence is greater than the curated BLASTP bitscore cutoff) (Alcock et al., 2020)

| **Antibiotic Resistance Ontology Term** | **AMR (antimicrobial resistance) Gene Family** | **Drug Class** | **Resistance Mechanism** | **% Identity of Matching Region** | **% Length of Reference Sequence** |
| --- | --- | --- | --- | --- | --- |
| adeF | resistance-nodulation-cell division (RND) antibiotic efflux pump | fluoroquinolone antibiotic, tetracycline antibiotic | antibiotic efflux | 63.31 | 100.38 |
| adeF | resistance-nodulation-cell division (RND) antibiotic efflux pump | fluoroquinolone antibiotic, tetracycline antibiotic | antibiotic efflux | 66.86 | 100 |
| adeF | resistance-nodulation-cell division (RND) antibiotic efflux pump | fluoroquinolone antibiotic, tetracycline antibiotic | antibiotic efflux | 43.49 | 97.45 |
| Acinetobacter baumannii AbaQ | major facilitator superfamily (MFS) antibiotic efflux pump | fluoroquinolone antibiotic | antibiotic efflux | 72.96 | 101.38 |
| FosA | fosfomycin thiol transferase | fosfomycin | antibiotic inactivation | 70.59 | 102.22 |
| adeF | resistance-nodulation-cell division (RND) antibiotic efflux pump | fluoroquinolone antibiotic, tetracycline antibiotic | antibiotic efflux | 41.09 | 99.06 |
| Pseudomonas aeruginosa soxR | ATP-binding cassette (ABC) antibiotic efflux pump, major facilitator superfamily (MFS) antibiotic efflux pump, resistance-nodulation-cell division (RND) antibiotic efflux pump | fluoroquinolone antibiotic, cephalosporin, glycylcycline, penam, tetracycline antibiotic, acridine dye, rifamycin antibiotic, phenicol antibiotic, triclosan, disinfecting agents and intercalating dyes | antibiotic target alteration, antibiotic efflux | 69.06 | 96.15 |

**Table S6:** Virulence factors identified in the SCA7 genome using Virulence Factor Database (VFDB Version 2019; Liu et al., 2019)

| **Virulence factors** | **related genes** | **SCA7 gene ID** |
| --- | --- | --- |
| **Adherence** |  |  |
| Flagella | *flaG* | KBP52_10460 |
| Flagella | *fleN* | KBP52_10285 |
| Flagella | *fleQ* | KBP52_10440 |
| Flagella | *fleR* | KBP52_10430 |
| Flagella | *fleS* | KBP52_10435 |
| Flagella | *flgA* | KBP52_25975 |
| Flagella | *flgB* | KBP52_25990 |
| Flagella | *flgC* | KBP52_25995 |
| Flagella | *flgD* | KBP52_26000 |
| Flagella | *flgE* | KBP52_26005 |
| Flagella | *flgF* | KBP52_10590 |
| Flagella | *flgG* | KBP52_10585 |
| Flagella | *flgH* | KBP52_10580 |
| Flagella | *flgI* | KBP52_10575 |
| Flagella | *flgJ* | KBP52_10570 |
| Flagella | *flgK* | KBP52_10565 |
| Flagella | *flgL* | KBP52_10560 |
| Flagella | *flgM* | KBP52_25970 |
| Flagella | *flgN* | KBP52_25965 |
| Flagella | *flhA* | KBP52_10295 |
| Flagella | *flhB* | KBP52_10335 |
| Flagella | *flhF* | KBP52_10290 |
| Flagella | *fliA* | KBP52_10280 |
| Flagella | *fliC* | KBP52_10465 |
| Flagella | *fliD* | KBP52_10455 |
| Flagella | *fliE* | KBP52_10425 |
| Flagella | *fliF* | KBP52_10420 |
| Flagella | *fliG* | KBP52_10415 |
| Flagella | *fliH* | KBP52_10410 |
| Flagella | *fliI* | KBP52_10405 |
| Flagella | *fliJ* | KBP52_10400 |
| Flagella | *fliK* | KBP52_10375 |
| Flagella | *fliL* | KBP52_10370 |
| Flagella | *fliM* | KBP52_10365 |
| Flagella | *fliN* | KBP52_10360 |
| Flagella | *fliO* | KBP52_10355 |
| Flagella | *fliP* | KBP52_10350 |
| Flagella | *fliQ* | KBP52_10345 |
| Flagella | *fliR* | KBP52_10340 |
| Flagella | *fliS* | KBP52_10450 |
| Flagella | *fliT* | KBP52_10445 |
| Flagella | *motA* | KBP52_15920 |
| Flagella | *motB* | KBP52_15915 |
| Flagella | *motC* | KBP52_10255 |
| Flagella | *motD* | KBP52_10250 |
| Flagella | *motY* | KBP52_24640 |
| LPS O-antigen (P. aeruginosa) | *--* | KBP52_26960 |
|  |  | KBP52_27025 |
| Type IV pili biosynthesis | *fimU* | KBP52_22820 |
|  |  | KBP52_22825 |
| Type IV pili biosynthesis | *fimV* | KBP52_07900 |
| Type IV pili biosynthesis | *pilA* | KBP52_22985 |
| Type IV pili biosynthesis | *pilB* | KBP52_22980 |
| Type IV pili biosynthesis | *pilC* | KBP52_07365 |
|  |  | KBP52_22975 |
| Type IV pili biosynthesis | *pilD* | KBP52_22970 |
| Type IV pili biosynthesis | *pilE* | KBP52_22850 |
| Type IV pili biosynthesis | *pilF* | KBP52_24185 |
| Type IV pili biosynthesis | *pilM* | KBP52_16435 |
| Type IV pili biosynthesis | *pilN* | KBP52_16430 |
| Type IV pili biosynthesis | *pilO* | KBP52_16425 |
| Type IV pili biosynthesis | *pilP* | KBP52_16420 |
| Type IV pili biosynthesis | *pilQ* | KBP52_16415 |
| Type IV pili biosynthesis | *pilR* | KBP52_07180 |
|  |  | KBP52_22865 |
| Type IV pili biosynthesis | *pilS* | KBP52_22870 |
| Type IV pili biosynthesis | *pilT* | KBP52_20435 |
| Type IV pili biosynthesis | *pilU* | KBP52_04810 |
| Type IV pili biosynthesis | *pilV* | KBP52_22830 |
| Type IV pili biosynthesis | *pilW* | KBP52_22835 |
| Type IV pili biosynthesis | *pilX* | KBP52_22840 |
| Type IV pili biosynthesis | *pilY1* | KBP52_22845 |
| Type IV pili biosynthesis | *pilZ* | KBP52_26525 |
| Type IV pili twitching motility related proteins | *chpA* | KBP52_20505 |
| Type IV pili twitching motility related proteins | *chpC* | KBP52_20510 |
| Type IV pili twitching motility related proteins | *pilG* | KBP52_20485 |
| Type IV pili twitching motility related proteins | *pilH* | KBP52_20490 |
| Type IV pili twitching motility related proteins | *pilI* | KBP52_20495 |
| Type IV pili twitching motility related proteins | *pilJ* | KBP52_20500 |
| **Antiphagocytosis** |  |  |
| Alginate biosynthesis | *alg44* | KBP52_13425 |
| Alginate biosynthesis | *alg8* | KBP52_13420 |
| Alginate biosynthesis | *algA* | KBP52_13470 |
| Alginate biosynthesis | *algC* | KBP52_19325 |
| Alginate biosynthesis | *algD* | KBP52_13415 |
| Alginate biosynthesis | *algE* | KBP52_13435 |
| Alginate biosynthesis | *algF* | KBP52_13465 |
| Alginate biosynthesis | *algG* | KBP52_13440 |
| Alginate biosynthesis | *algI* | KBP52_13455 |
| Alginate biosynthesis | *algJ* | KBP52_13460 |
| Alginate biosynthesis | *algK* | KBP52_13430 |
| Alginate biosynthesis | *algL* | KBP52_13450 |
| Alginate biosynthesis | *algX* | KBP52_13445 |
| Alginate regulation | *algP/algR3* | KBP52_19650 |
| Alginate regulation | *algQ* | KBP52_19640 |
| Alginate regulation | *algR* | KBP52_19610 |
| Alginate regulation | *algU* | KBP52_11375 |
| Alginate regulation | *algW* | KBP52_13920 |
| Alginate regulation | *mucA* | KBP52_11370 |
| Alginate regulation | *mucB* | KBP52_11365 |
| Alginate regulation | *mucD* | KBP52_11355 |
| Alginate regulation | *mucP* | KBP52_12670 |
| **Iron uptake** |  |  |
| Pyochelin receptor | *fptA* | KBP52_00990 |
| Pyochelin | *pchD* | KBP52_00385 |
| Pyochelin | *pchF* | KBP52_00380 |
| Pyoverdine receptors | *fpvA* | KBP52_00130 |
|  |  | KBP52_00485 |
|  |  | KBP52_01340 |
|  |  | KBP52_02635 |
|  |  | KBP52_06465 |
|  |  | KBP52_08175 |
|  |  | KBP52_13550 |
|  |  | KBP52_13925 |
|  |  | KBP52_17820 |
|  |  | KBP52_18470 |
|  |  | KBP52_21040 |
| Pyoverdine | *pvdA* | KBP52_08125 |
| Pyoverdine | *pvdD* | KBP52_08195 |
| Pyoverdine | *pvdE* | KBP52_08170 |
| Pyoverdine | *pvdG* | KBP52_08200 |
| Pyoverdine | *pvdH* | KBP52_27565 |
| Pyoverdine | *pvdI* | KBP52_08185 |
|  |  | KBP52_08195 |
|  |  | KBP52_17795 |
| Pyoverdine | *pvdL* | KBP52_27520 |
| Pyoverdine | *pvdM* | KBP52_08155 |
| Pyoverdine | *pvdN* | KBP52_08160 |
| Pyoverdine | *pvdO* | KBP52_08165 |
| Pyoverdine | *pvdP* | KBP52_08150 |
| Pyoverdine | *pvdQ* | KBP52_02530 |
| Pyoverdine | *pvdS* | KBP52_27515 |
| Pyoverdine | *pvdY* | KBP52_27510 |
| Alkaline protease | *aprA* | KBP52_04060 |
|  |  | KBP52_06410 |
| **Quorum sensing** |  |  |
| Acylhomoserine lactone synthase (in PGAP identified as 1-acyl-sn-glycerol-3-phosphate acyltransferase) | *hdtS* | KBP52_18405 |
| **Regulation** |  |  |
| GacS/GacA two-component system | *gacA* | KBP52_04730 |
| GacS/GacA two-component system | *gacS* | KBP52_26130 |
| Carbon storage regulator A | *csrA* | KBP52_07800 |
| **Secretion system** |  |  |
| Hcp secretion island-1 encoded type VI secretion system (H-T6SS) | *--* | KBP52_02720 |
| Hcp secretion island-1 encoded type VI secretion system (H-T6SS) | *icmF1* | KBP52_02765 |
| Hcp secretion island-1 encoded type VI secretion system (H-T6SS) | *vgrG1* | KBP52_02700 |
|  |  | KBP52_04330 |
|  |  | KBP52_16195 |
| P. syringae TTSS effectors | *hopAJ2* | KBP52_22275 |
| P. syringae TTSS effectors | *hopJ1* | KBP52_13785 |
| **Toxin** |  |  |
| Exolysin | *exlA* | KBP52_28365 |
| Exolysin | *exlB* | KBP52_28360 |
| Hydrogen cyanide production | *hcnA* | KBP52_29820 |
| Hydrogen cyanide production | *hcnB* | KBP52_29825 |
| Hydrogen cyanide production | *hcnC* | KBP52_29830 |
| TccC-type insecticidal toxins | *--* | KBP52_24930 |
|  |  | KBP52_24935 |
| **Invasion** |  |  |
| Invasion of brain endothelial cells (Ibes) | *ibeC* | KBP52_02655 |
| **Lipid and fatty acid metabolism** |  |  |
| Isocitrate lyase | *icl* | KBP52_29410 |
| **Stress adaptation** |  |  |
| Catalase-peroxidase | *katG* | KBP52_00315 |
| Catalase | *katA* | KBP52_21780 |
| Superoxide dismutase protein SodCI | *sodCI* | KBP52_06765 |

**Table S7**: Characteristics of the detected volatile organic compounds (VOCs). CAS: Chemical Abstracts Service, RT: retention time, I: Kovats retention index, m/z: mass to charge ratio

| Compound name | CAS  registry no. | RT | *I* | 1^st^ m/z | Absolute abundance [%] of 1st m/z |
| --- | --- | --- | --- | --- | --- |
|  |  |  |  |  |  |
| *octanal* | 124-13-0 | 11.1 | 991 | 41 | 100 |
| *(E)-1,4-undecadiene* | 55976-13-1 | 12.86 | 1079 | 54 | 100 |
| *1-undecene* | 821-95-4 | 12.969 | 1085 | 55 | 100 |
| *unknown* | - | 24.966 | 1653 | 97 | 100 |

# Supplementary Figures


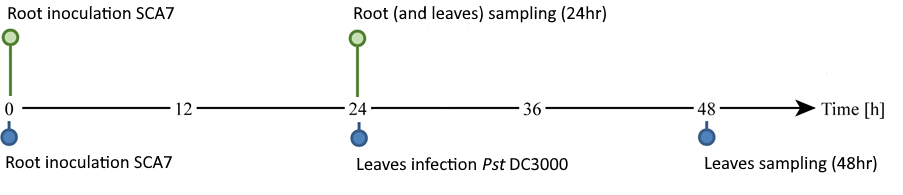
**Supplementary Figure 1:** Inoculation and harvesting time points of experiments with *A. thaliana* plants treated with SCA7 only (green) and in combination with the pathogenic *Pst* DC3000 (blue).


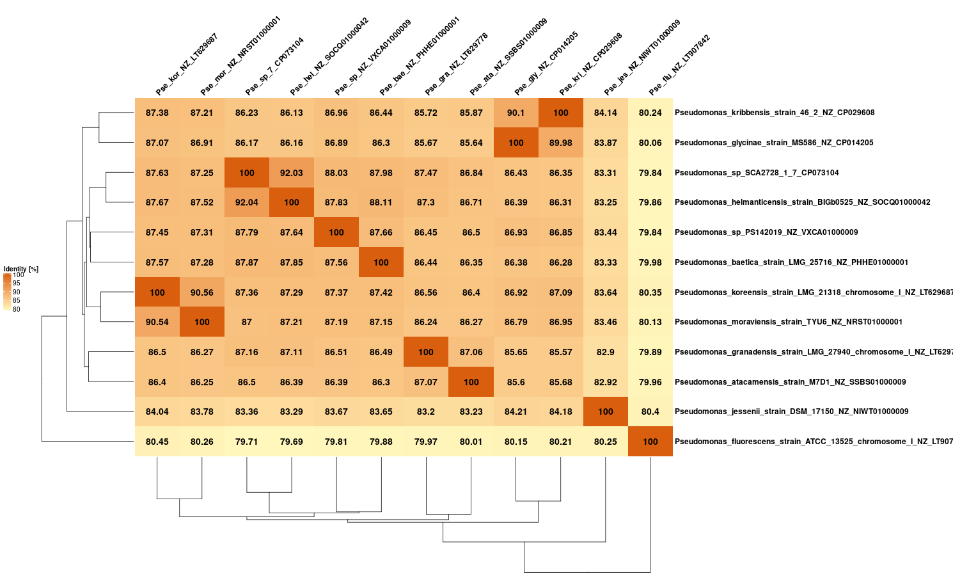


**Supplementary Figure 2**: Average Nucleotide Identity (ANI) Matrix of *Pseudomonas* sp. SCA7 (=SCA2728.1_7) based on 11 full closest related *Pseudomonas* type strain genomes. Data obtained with EDGAR Software.


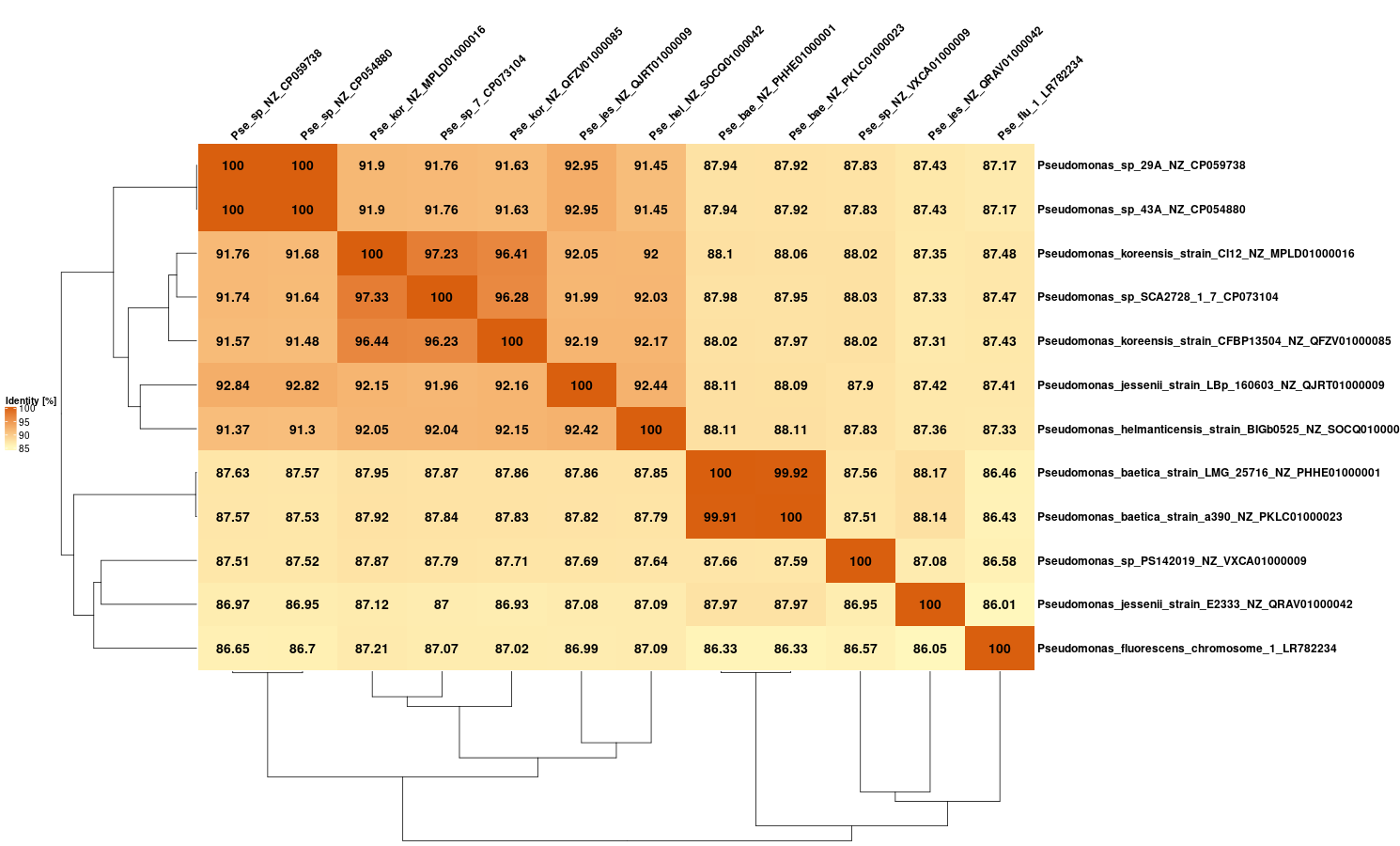


**Supplementary Figure 3**: Average Nucleotide Identity (ANI) Matrix of *Pseudomonas* sp. SCA7 based on 11 full closest related *Pseudomonas* genomes. Data obtained with EDGAR Software.


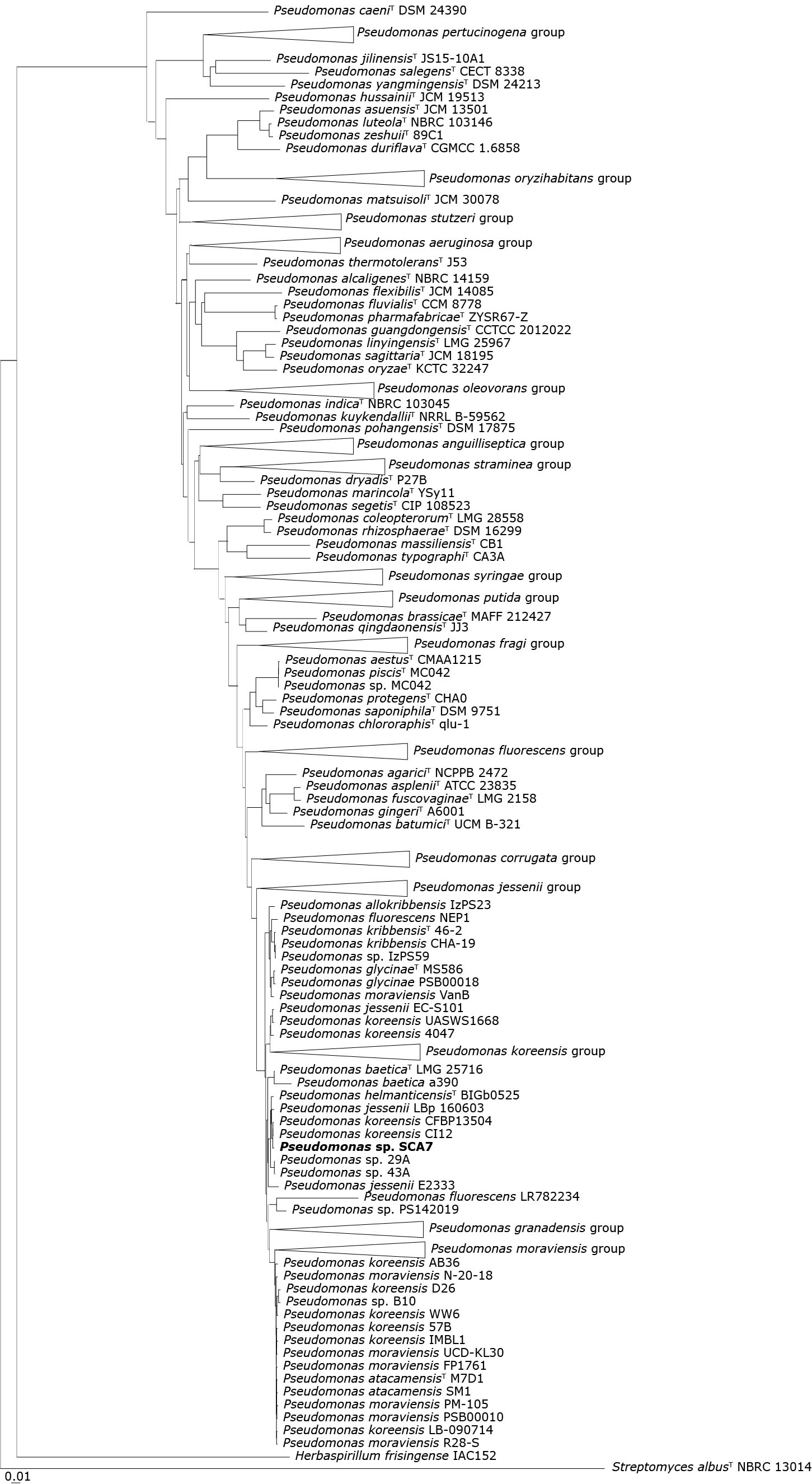


**Supplementary Figure 4**: Maximum-likelihood phylogenetic tree of the *Pseudomonas* genus generated with FastTree from 70 genes of the core genome. Triangles represent species groups according to Gomila et al. (2015). The scale bar represents nucleotide substitutions per site (0.01 scale = 1% nucleotide substitutions per site).


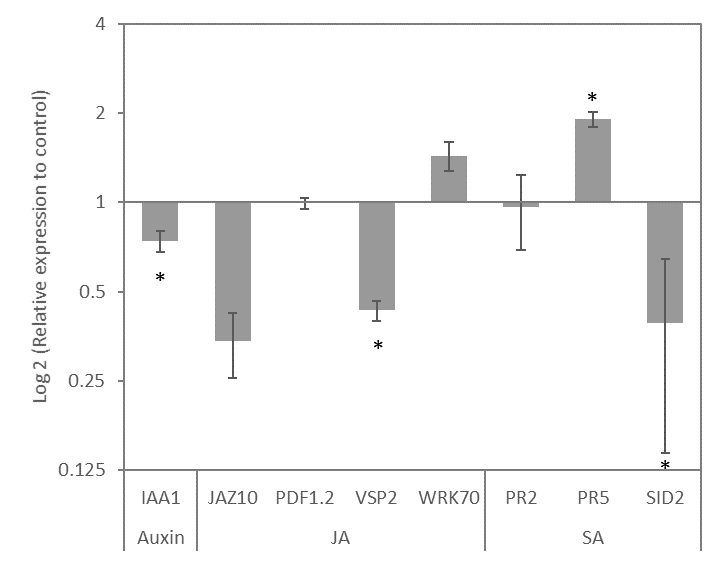


**Supplementary Figure 5**: Relative to control expression of marker genes after 24 h involved in auxin, nutrient uptake and defense responses after in *A. thaliana* seedling shoots inoculated with SCA7. Error bars indicate standard deviation. Asterisks indicate significant differences (p-value * < 0.05).
